# γ-tubulin mediates DNA double-strand break repair

**DOI:** 10.1242/jcs.262255

**Published:** 2025-03-26

**Authors:** Abhishikt David Solomon, Odjo G. Gouttia, Ling Wang, Songli Zhu, Feifei Wang, Yanqui Li, Mohammadjavad Paydar, Tadayoshi Bessho, Benjamin H. Kwok, Aimin Peng

**Affiliations:** ^1^ Division of Oral and Craniofacial Health Sciences, Adams School of Dentistry, University of North Carolina at Chapel Hill, Chapel Hill, NC 27599, USA; ^2^ Lineberger Comprehensive Cancer Center, University of North Carolina at Chapel Hill, Chapel Hill, NC 27599, USA; ^3^ Department of Oral Biology, University of Nebraska Medical Center, Lincoln, NE 68583, USA; ^4^ Institute for Research in Immunology and Cancer (IRIC), Département de médecine, Université de Montréal, Montréal H3C 3J7, Canada; ^5^ Eppley Institute for Research in Cancer and Allied Diseases, University of Nebraska Medical Center, Omaha, NE 68198, USA

**Keywords:** DNA repair, Double-strand break, γ-tubulin, Microtubule

## Abstract

Double-strand breaks (DSBs) in DNA pose a critical threat to genomic integrity, potentially leading to the onset and progression of various diseases, including cancer. Cellular responses to such lesions entail sophisticated repair mechanisms primarily mediated by non-homologous end joining (NHEJ) and homologous recombination (HR). Interestingly, the efficient recruitment of repair proteins and completion of DSB repair likely involve complex, inter-organelle communication and coordination of cellular components. In this study, we report a role of γ-tubulin in DSB repair. γ-tubulin is a major microtubule nucleation factor governing microtubule dynamics. We show that γ-tubulin is recruited to the site of DNA damage and is required for efficient DSB repair via both NHEJ and HR. Suppression of γ-tubulin impedes DNA repair and exacerbates DNA damage accumulation. Furthermore, γ-tubulin mediates the mobilization and formation of DNA damage foci, which serve as repair centers, thereby facilitating the recruitment of HR and NHEJ repair proteins on damaged chromatin. Finally, pharmacological inhibition of γ-tubulin enhances the cytotoxic effect of DNA-damaging agents, consistent with the DNA repair function of γ-tubulin, and underscoring the potential of its therapeutic intervention in cancer therapy.

## Teaser

**Summary:** γ-tubulin is recruited to DNA damage sites, mediates DNA break mobility and facilitates DNA repair. This study provides proof of principle for therapeutic targeting of γ-tubulin in cancer therapy.

## INTRODUCTION

DNA damage presents a significant risk to genome integrity, prompting cells to employ a spectrum of sophisticated mechanisms of the DNA damage response. These mechanisms enable cells to detect DNA damage, initiate signaling cascades and activate specific DNA repair machinery to remove various forms of DNA damage (Harper and Elledge, 2007; Jackson and Bartek, 2009; Sancar et al., 2004). Among different types of DNA lesions, double-strand breaks (DSBs) stand out as particularly perilous and deleterious, capable of inducing severe genomic instability and elevating the risk of diseases such as cancer, neurodegenerative diseases, premature aging and immunodeficiencies (Khanna and Jackson, 2001; Trenner and Sartori, 2019). DSBs can arise from a variety of sources including environmental stress, reactive oxygen species, ionizing radiation (IR) and many other exogenous or endogenous agents. These DSBs need to be efficiently repaired to mitigate potential adverse outcomes. In contrast, abundant DSBs are deliberately induced in the context of radiotherapy and chemotherapy of patients with cancer, to eliminate malignant cells. In this scenario, the repair of DSBs confers cell survival and thus presents an attractive drug target for enhancing treatment efficacy.

DSBs can be repaired via non-homologous end joining (NHEJ) or homologous recombination (HR). Both these pathways involve numerous factors and complex regulatory mechanisms to mediate DSB sensing and repair. NHEJ operates by directly ligating broken DSB ends, independent of a homologous template strand, making it a rapid and versatile repair option. In this process, the Ku70 (also known as X-ray repair cross-complementing protein 6 or XRCC6) and Ku80 (XRCC5) subunits of the DNA-dependent protein kinase complex (DNA-PK) promptly recognize and bind to DSB ends, initiating the recruitment and activation of the catalytic subunit (DNA-PKcs, also known as PRKDC). The subsequent assembly of factors such as artemis (also known as DCLRE1C), polymerase λ (*POLL*) and others facilitates end processing, enabling the DNA ligase IV (LIG4)–XRCC4–XRCC4-like factor (XLF, also known as NHEJ1) complex to seal the breaks (Chiruvella et al., 2013; Davis and Chen, 2013; Lieber, 2010). Conversely, HR predominantly operates during the S and G2 phases of the cell cycle, after chromatin DNA replication, leveraging an intact homologous template strand for precise repair. Here, DSB ends undergo controlled resection to generate 3′ single-stranded DNA (ssDNA) overhangs. These ssDNA regions are initially safeguarded by replication protein A (RPA) and subsequently coated by recombinase RAD51. RAD51-mediated strand invasion and homology-directed repair facilitate faithful restoration of the damaged DNA sequence (Heyer et al., 2010; Jasin and Rothstein, 2013).

γ-tubulin has been intensively investigated as a microtubule (MT) nucleator and is one of the key proteins that regulate MT dynamics (Aher et al., 2024; Kollman et al., 2011; Oakley et al., 2015). γ-tubulin resides within MT-organizing centers (MTOCs) or centrosomes, where it forms the γ-tubulin small complex (γ-TUSC) with GCPs (γ-tubulin complex proteins). Nuclear localization and functions of γ-tubulin have been implicated in recent studies. For example, γ-tubulin is phosphorylated at the G1/S transition stage of the cell cycle, facilitating its accumulation in the nucleus (Eklund et al., 2014; Hoog et al., 2011). The nuclear-localized γ-tubulin interacts with E2 promoter-binding factor (E2F) to modulate its transcriptional activity (Hoog et al., 2011) and facilitates the chromatin recruitment of proliferating cellular nuclear antigen (PCNA) (Corvaisier et al., 2021). It has also been shown that DNA damage induces the colocalization between γ-tubulin and RAD51, implying a link between γ-tubulin and HR (Lesca et al., 2005).

In this study, we identify γ-tubulin as a DSB-binding protein *in vitro* and establish it as a protein recruited to DNA damage sites within the cell. Our studies further suggest a model in which γ-tubulin mediates the mobilization of DSBs and formation of repair centers, thereby facilitating the recruitment of repair proteins and promoting DNA repair. Conversely, γ-tubulin depletion hinders DNA repair, resulting in DNA damage accumulation and hypersensitivity to DNA damage.

## RESULTS

### γ-tubulin associates with DSB-mimicking DNA *in vitro*, and is recruited to DNA damage sites in cells

**Fig. 1.**
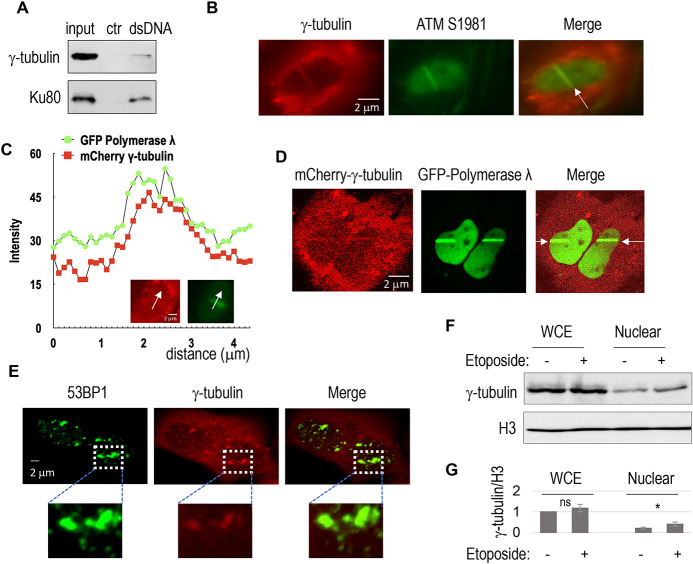
**γ-tubulin associates with DSB-mimicking DNA *in vitro*, and is recruited to DNA damage sites in cells.** (A) Pulldown with biotin-labeled dsDNA (500 bp with free ends, as described in Zhu et al., 2020) mimicking double-strand breaks (DSBs) was performed using HeLa cell lysates. A control (ctr) pulldown was performed using blank beads. The input, control pulldown and dsDNA pulldown samples were analyzed by immunoblotting (IB). *n*=3 independent experiments. (B) SCC38 cells were laser-microirradiated and analyzed by immunofluorescence for γ-tubulin and phospho-ATM S1981, as described in the Materials and Methods. The images were taken 3 min post laser treatment. The region of laser microirradiation is denoted by the white arrow. *n*=3 independent experiments. (C,D) HeLa cells with transient expression of mCherry–γ-tubulin and GFP–polymerase λ were laser microirradiated after pre-sensitization and imaged, as described in the Materials and Methods. The images were taken 5 min post laser treatment. Colocalization of mCherry–γ-tubulin and GFP–polymerase λ after laser microirradiation was quantified in panel C and shown in panel D. Arrows in the inset images in C indicate the points chosen for colocalization analysis. The regions of laser microirradiation are denoted by white arrows in D. *n*=3 independent experiments. (E) HeLa cells were treated with etoposide (1 µM, 10 min) and analyzed by immunofluorescence for γ-tubulin and 53BP1. The colocalization is shown. *n*=3 independent experiments. (F) Whole-cell extracts (WCE) and nuclear fractions were isolated from HeLa cells with or without etoposide treatment (1 µM, 3 h), as described in the Materials and Methods, and analyzed by IB for γ-tubulin and histone H3. (G) The IB results in panel F were quantified. Band intensities of γ-tubulin were normalized to those of histone H3. *n*=3 independent experiments. Bars show mean±s.d. Two-tailed unpaired Student's *t*-test was performed; ns, not significant; *P<0.05.

In our previous investigation, we characterized the kinesin motor protein Kif2C as a DSB-binding protein within *Xenopus* egg extracts (Zhu et al., 2020). Concurrently, we identified γ-tubulin as another potential DSB-associated protein (Fig. S1A). To confirm this finding, we performed a pull-down assay in HeLa cell lysates using a dsDNA fragment harboring free DSB ends. Interestingly, γ-tubulin was recovered in association with this DSB-containing DNA template (Fig. 1A). Despite its predominant localization in the cellular cytosol, nuclear γ-tubulin was recruited to sites of DNA damage following laser microirradiation, colocalizing with the established DNA damage markers phospho-ATM (Ser 1981) and poly-ADP-ribose (PAR) (Fig. 1B; Fig. S1B). The specific immunofluorescence signal of γ-tubulin was confirmed by siRNA-mediated γ-tubulin depletion (Fig. S1C). Furthermore, mCherry-tagged recombinant γ-tubulin exhibited recruitment to laser-microirradiated sites, akin to the localization pattern observed with GFP-tagged DNA polymerase λ (Fig. 1C,D). Consistent recruitment of γ-tubulin to DNA damage sites was also observed using another distinct system of laser microirradiation and imaging (Fig. 1D; Fig. S1D,E). Analysis of intranuclear foci formation showed that γ-tubulin colocalized with DNA damage-induced foci of 53BP1 (also known as TP53BP1), a marker of DNA damage, particularly DSBs (Fig. 1E, Movies 1–4).

Inhibition of ATM/ATR or DNA-PKcs did not prevent γ-tubulin recruitment to laser-induced DNA damage sites (Fig. S2), suggesting that γ-tubulin recruitment occurs through a mechanism independent of these upstream kinases. Notably, biochemical fractionation experiments revealed that a small fraction of γ-tubulin is localized within the cell nucleus under normal conditions. Following DNA damage, this nuclear localization was markedly increased (Fig. 1F,G). Collectively, these findings identified γ-tubulin as a DNA damage response protein that was rapidly recruited to sites of DNA damage.

### γ-tubulin associates with DNA repair proteins

**Fig. 2.**
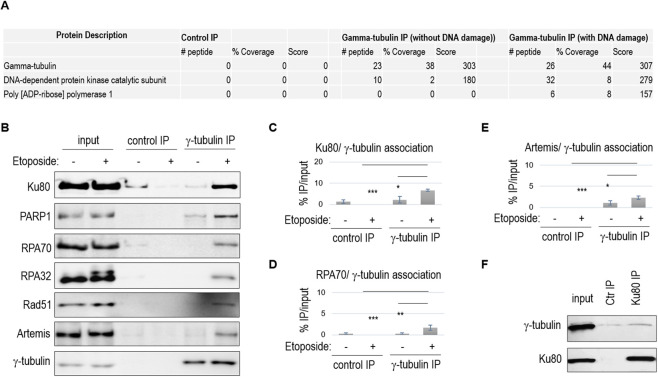
**γ-tubulin associates with DNA repair proteins.** (A) As described in the Materials and Methods, γ-tubulin immunoprecipitation (IP) was performed in lysates of HeLa cells that were untreated or treated with bleomycin (20 µM, 3 h) to induce DNA damage. The γ-tubulin IP products and control IP products with blank beads were subjected to proteomic analysis for identification of associated proteins. Poly-ADP-ribose polymerase 1 (PARP1) and the catalytic subunit (DNA-PKcs) of the DNA-dependent protein kinase complex were identified as γ-tubulin-associated proteins, as shown by the numbers of identified peptides, percentage of coverage and Mascot scores (via RAW MS data to MGF conversion and Mascot Server analysis). (B) γ-tubulin IP was performed in HeLa cells with or without etoposide. The input (10%), control IP using blank beads and γ-tubulin IP products were analyzed by IB for the indicated proteins. (C–E) Quantification of control or γ-tubulin IP products, as shown in panel B, was shown as the percentage of the input. Bars show mean±s.d. Two-tailed unpaired Student's *t*-test was performed from >3 independent experiments; *P<0.05; **P<0.01; ***P<0.001. (F) Ku80 IP was performed in HeLa cells with bleomycin treatment. The input, control IP using blank beads, and Ku80 IP products were analyzed by IB. *n*=3 independent experiments.

We used proteomic analysis to identify proteins that co-immunoprecipitated with γ-tubulin in cell lysates. Notably, two established DNA repair proteins, namely, the pivotal NHEJ factor DNA-PKcs and the early DNA damage responder poly-ADP-ribose polymerase 1 (PARP1), were identified as γ-tubulin-associated proteins (Fig. 2A). Validation of γ-tubulin associations with Ku80 and PARP1 was carried out by immunoblotting of the immunoprecipitated products (Fig. 2B,C). These associations were detected in the absence of DNA damage but were notably enhanced post DNA damage induction (Fig. 2B). Conversely, Ku80 immunoprecipitation also yielded a portion of γ-tubulin (Fig. 2F), indicating a reciprocal association between these proteins. Notably, although not identified in the proteomic analysis, several other factors of HR and NHEJ, including RAD51, RPA70 (also known as RPA1), RPA32 (also known as RPA2) and artemis, were also detected in the products of γ-tubulin immunoprecipitation, with increased abundance after DNA damage (Fig. 2C–E).

### γ-tubulin depletion or inhibition leads to accumulation of endogenous DNA damage and delays DNA repair

**Fig. 3.**
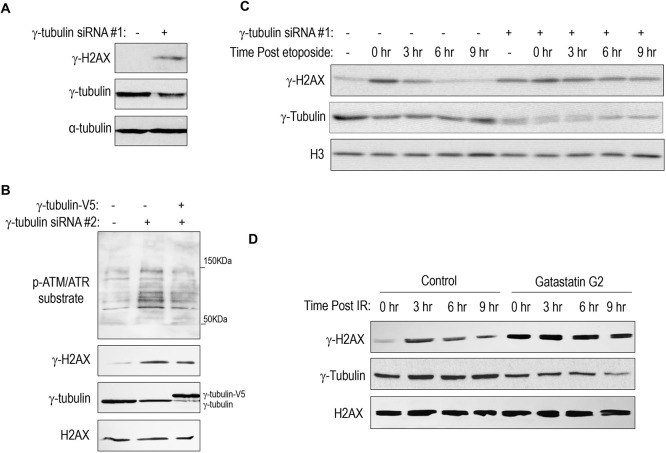
**Inhibition of γ-tubulin leads to accumulation of endogenous DNA damage and delayed DNA repair.** (A) HeLa cells were treated with control or γ-tubulin siRNA for 1 day. The cells were harvested and analyzed by IB. *n*=3 independent experiments. (B) HeLa cells were treated with control or γ-tubulin siRNA, and with or without transfection of a γ-tubulin-expressing vector, for 1 day. The cells were harvested and analyzed by IB. *n*=3 independent experiments. (C) For the repair kinetics analysis, HeLa cells with control or γ-tubulin siRNA were treated with 1 μM etoposide for 1 h, followed by repair/recovery for 0, 3, 6 and 9 h. The cells were harvested and analyzed by IB. *n*=3 independent experiments. (D) HeLa cells were treated with or without 6 μM gatastatin G2 and irradiated (IR), followed by repair/recovery for 0, 3, 6 and 9 h. The cells were harvested and analyzed by IB. *n*=3 independent experiments.

The recruitment of γ-tubulin to DNA damage sites, along with its association with repair proteins, prompted an investigation into the functional significance of γ-tubulin in DNA repair processes. Depletion of γ-tubulin using an siRNA targeting the coding sequence of the *TUBG1* gene resulted in an augmentation of γ-H2AX levels (Fig. 3A), indicative of increased accumulation of endogenous DNA damage. Consistently, knockdown using another siRNA targeting the 3ʹ untranslated region of *TUBG1* also led to elevated γ-H2AX levels and subsequent ATM/ATR-mediated protein phosphorylation, a hallmark signaling event triggered by DNA damage (Fig. 3B). Moreover, re-expression of recombinant HA-tagged γ-tubulin partially rescued the accumulation of γ-H2AX and ATM/ATR-mediated protein phosphorylation (Fig. 3B). The knockdown of γ-tubulin similarly increased DNA damage in cells synchronized in the G1 phase through CDK4/CDK6 inhibition and in cells synchronized in the S phase via thymidine arrest (Fig. S3A).

Depletion of γ-tubulin not only elevated endogenous γ-H2AX levels but also impeded DNA repair kinetics. γ-tubulin knockdown delayed the repair of DNA damage caused by etoposide treatment, as indicated by the sustained γ-H2AX signal observed over 3–9 h (Fig. 3C). Similarly, although IR-induced DNA damage, measured by γ-H2AX levels, was efficiently repaired within 3–6 h in control cells, the damage persisted beyond 9 h in γ-tubulin-suppressed cells (Fig. S3B). Both the induction of endogenous DNA damage and the impaired DNA repair following etoposide treatment, due to γ-tubulin depletion, were further confirmed at the single-cell level through quantification of 53BP1 foci (Fig. S4).

Next, we assessed whether pharmacological inhibition of γ-tubulin activity would replicate the observed effects of its depletion. To this end, we used gatastatin G2, a specific inhibitor targeting γ-tubulin activity and impeding MT nucleation mediated by γ-tubulin (Chinen et al., 2015), and 3,4′,5-trimethoxy-trans-stilbene (MR-3), a methylated analog of resveratrol known to target γ-tubulin (Traversi et al., 2019). Notably, treatment with both gatastatin G2 and MR-3 resulted in elevated endogenous γ-H2AX levels, along with sustained γ-H2AX levels following IR treatment, indicative of impaired DNA repair processes (Fig. 3D; Fig. S5).

### γ-tubulin promotes DSB repair via both NHEJ and HR

**Fig. 4.**
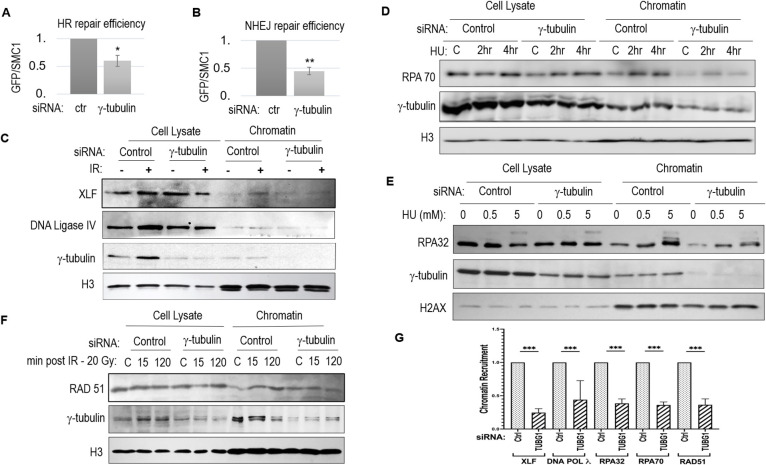
**γ-tubulin suppression reduces DSB repair efficacy.** (A,B) Chromosome-integrated, I-SceI-induced homologous recombination (HR) (A) or non-homologous end joining (NHEJ) (B) assays were performed as described in the Materials and Methods. The reporter cells, HeLa-Dr or U2OS-EJ5, were transfected with control (ctr) or γ-tubulin siRNA. I-SceI endonuclease was expressed using a lentiviral vector. Following 48 h incubation, DNA repair was measured by IB to assess GFP expression relative to SMC1 expression. The GFP/SMC1 ratio in γ-tubulin-depleted cells was normalized to that in control cells for relative repair efficiency. The mean±s.d. values, calculated from three independent experiments, are shown. Statistical significance was analyzed using an unpaired two-tailed Student's *t*-test; *P<0.05; **P<0.01. (C) HeLa cells treated with control or γ-tubulin siRNA were irradiated with 20 Gy of X-ray irradiation (IR), followed by repair/recovery for 30 min. Chromatin fractionation was performed as described in the Materials and Methods. Whole-cell lysates and chromatin-enriched fractions were analyzed by IB. *n*=5 independent experiments. (D) HeLa cells were treated with control or γ-tubulin siRNA. Cells were then control treated (‘C’) or treated with hydroxyurea (HU, 5 mM) for 2 or 4 h. Chromatin fractionation was performed. Whole-cell lysates and chromatin-enriched fractions were analyzed by IB. *n*=5 independent experiments. (E) HeLa cells were treated with control or γ-tubulin siRNA. Cells were then control treated (‘C’) or treated with HU at the indicated concentrations for 3 h. Chromatin fractionation was performed. Whole-cell lysates and chromatin-enriched fractions were analyzed by IB. *n*=5 independent experiments. (F) HeLa cells were treated with or without γ-tubulin siRNA. Cells were then control treated (‘C’) or treated with 20 Gy IR. Cells were incubated post IR for the indicated durations (in minutes) and then subjected to chromatin fractionation. Whole-cell lysates and chromatin-enriched fractions were analyzed by IB. *n*=5 independent experiments. (G) The chromatin recruitment of NHEJ and HR repair proteins, normalized to that of H3, was analyzed for control or γ-tubulin siRNA-treated groups. *n*=5 independent experiments. Bars show mean±s.d. Statistical analysis was performed using two-tailed unpaired Student's *t*-test; ***P<0.001.

We used endonuclease I-SceI-induced NHEJ and HR reporter systems, as described previously (Gunn and Stark, 2012), to evaluate the impact of γ-tubulin depletion on these DSB repair pathways. Using a *TUBG1*-specific siRNA, we observed a significant suppression of both NHEJ and HR pathways upon γ-tubulin depletion (Fig. 4A,B). Consistent with these repair deficiencies, we observed reduced recruitment of NHEJ and HR repair factors to damaged chromatin. Specifically, in HeLa cells subjected to IR treatment, the chromatin-loading of the DNA ligase IV–XLF complex was notably reduced following γ-tubulin depletion (Fig. 4C; Fig. S2). Similarly, the chromatin enrichment of HR factors such as RPA70, RPA32 and RAD51, post IR or hydroxyurea (HU) treatment inducing replication stress and HR, was diminished in cells lacking γ-tubulin (Fig. 4D–F; Fig. S6). Quantitative analysis revealed a general reduction of 50–60% in the chromatin recruitment of DSB repair factors upon γ-tubulin depletion (Fig. 4G).

### γ-tubulin mediates the mobilization and formation of DNA damage foci

**Fig. 5.**
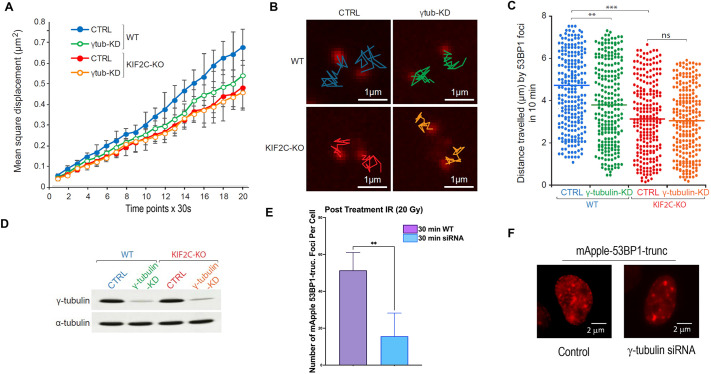
**γ-tubulin depletion reduces the mobilization and formation of DNA damage foci.** (A–D) Wild-type (WT) or Kif2C knockout (KO) U2OS cells were treated with control or γ-tubulin siRNA (knockdown, KD). (A) Mean square displacement measurements of EGFP–53BP1 foci were carried out as described in the Materials and Methods, and are shown. (B) Examples of 10 min mobility traces of EGFP–53BP1 are shown. (C) The measured distances traveled by individual 53BP1 foci are shown. The central bar shows the mean. Statistical analysis was performed using two-tailed unpaired Student's *t*-test; ns, not significant; **P<0.01; ***P<0.001. (D) The depletion of γ-tubulin was confirmed by IB. EGFP–53BP1 was transiently transfected, and expressed at a moderate level, as observed by immunofluorescence (Fig. S7C), relative to endogenous 53BP1. (E,F) HeLa cells expressing mApple–53BP1 were treated with control or γ-tubulin siRNA. Cells were irradiated with 20 Gy of X-ray irradiation (IR), followed by 30 min incubation. 53BP1 foci are shown in panel F and quantified in panel E (*n*>100). All data were collected from at least three independent experimental sets as mean±s.d. **P<0.01 by unpaired two-tailed Student's *t*-test.

The MT network mediates the movement of various cellular components, including chromatin. In previous work, we demonstrated the recruitment of the kinesin motor protein Kif2C to DNA damage sites, and its role in facilitating the mobilization of DSBs marked by DNA damage-induced 53BP1 foci (Zhu et al., 2020). Intriguingly, we observed a reduction in the mobility of 53BP1 foci upon depletion of γ-tubulin, as measured by both mean square displacement (Fig. 5A,B) and distance traveled by 53BP1 foci (Fig. 5C). Simultaneous depletion of both γ-tubulin and Kif2C failed to exacerbate the reduction in foci mobility, suggesting that these factors operate in a coordinated manner within the same pathway (Fig. 5A–D).

Previous studies have demonstrated that DNA damage breaks cluster to form nuclear macrodomains, visually represented as DNA damage foci (Lisby and Rothstein, 2004; Neumaier et al., 2012). This clustering process is presumed to rely on the mobilization of DNA breaks, potentially involving γ-tubulin. Indeed, depletion of γ-tubulin significantly attenuated the formation of 53BP1 foci at both 15 min and 30 min following DNA damage induction (Fig. 5E,F; Fig. S7A,B). Importantly, this reduction in foci formation was not attributed to diminished levels of DNA damage, as γ-tubulin suppression resulted in even higher levels of DNA damage accumulation. Rather, it likely reflects a deficiency in the clustering of DNA damage events.

### Pharmacological inhibition of γ-tubulin leads to DNA damage hypersensitivity

**Fig. 6.**
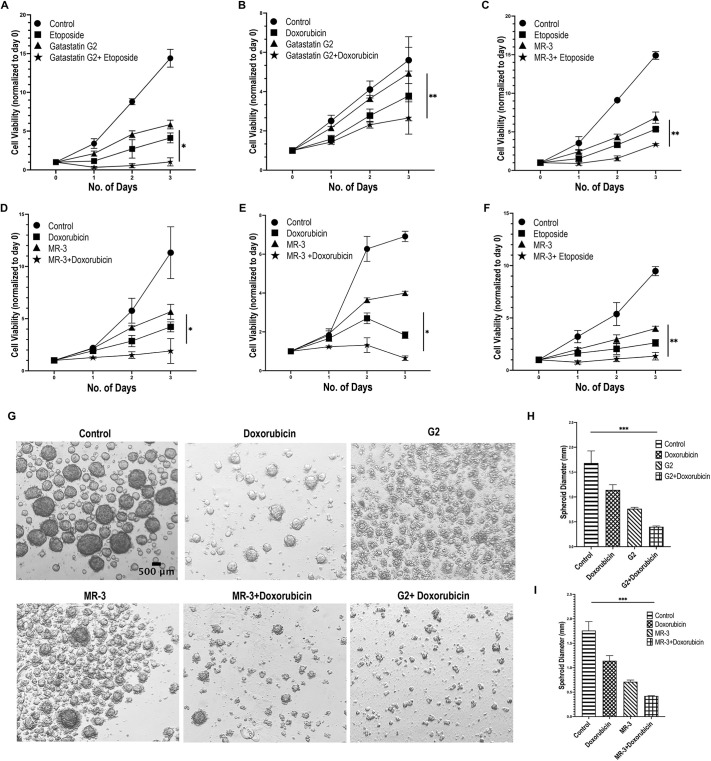
**Inhibition of γ-tubulin enhances tumor cell responses to DNA damage.** (A–D) HeLa cells were treated with control, etoposide (2 µM), gatastatin G2 (2 µM), doxorubicin (3 µM), MR-3 (15 µM) and combinations of drugs, as indicated. Cell numbers were quantified as described in the Materials and Methods. Relative cell viability was calculated by normalizing the final cell count relative to the count recorded on the initial day. The displayed results depict the mean±s.d. derived from at least three distinct experiments. *P<0.05; **P<0.01; two-way ANOVA with Tukey's multiple post hoc test. (E,F) SCC38 cells were treated with control, etoposide (3 µM), doxorubicin (4 µM), MR-3 (15 µM) and combinations of drugs, as indicated. Cell numbers were quantified, and relative cell viability was calculated by normalizing the final cell count relative to the count recorded on the initial day. The displayed results depict the mean±s.d. derived from at least three distinct experiments. *P<0.05; **P<0.01; one-way ANOVA with Dunnett's multiple post hoc test. (G–I) SCC38 cells were maintained under anchorage-independent culture conditions. Cells were then incubated with gatastatin G2 (3 µM), MR-3 (15 µM), doxorubicin (4 µM) and combinations of drugs, as indicated. (G) Representative images of spheroid formation. (H,I) The relative diameters of spheroids in treated groups were normalized to that of the control. The data were analyzed from at least three independent experimental sets using one-way ANOVA with Dunnett's multiple post hoc test and are depicted as mean±s.e.m. ***P<0.001.

The involvement of γ-tubulin in DNA repair underscores its potential as a therapeutic target to enhance the efficacy of DNA-damaging agents. As shown in this study, two inhibitors of γ-tubulin, gatastatin G2 and MR-3, delayed DNA repair processes and exacerbated DNA damage accumulation. Building upon this, we further reveal that both MR-3 and gatastatin G2 enhanced the cytotoxic effects of etoposide and doxorubicin and further suppressed cell viability in HeLa cells (Fig. 6A–D). Similar results were seen also in the SCC38 head and neck cancer cell line (Fig. 6E,F). Moreover, we extended our investigation to evaluate the effects of these drug combinations on anchorage-independent cell spheroid growth (Fig. 6G–I). Consistently, combining γ-tubulin inhibition with DNA damage resulted in reduced spheroid diameter and disrupted three-dimensional tumor cell growth. These findings collectively highlight the potential of targeting γ-tubulin in combination with DNA-damaging agents for enhanced therapeutic outcomes in cancer treatment.

## DISCUSSION

In our current study, we demonstrated the association of γ-tubulin with a DNA substrate mimicking DSBs *in vitro*, along with its co-immunoprecipitation with DNA repair proteins. Despite its predominant cytoplasmic localization, a fraction of γ-tubulin was recruited to DNA damage sites induced by laser microirradiation in the nucleus, where it colocalized with established DNA damage factors following treatment with damaging agents. Depletion of γ-tubulin resulted in elevated levels of endogenous DNA damage and impaired DNA repair. Consistently, γ-tubulin depletion attenuated both HR and NHEJ, as measured by established reporter assays. Our investigations revealed a reduction in the mobility and formation of DNA damage foci in γ-tubulin-depleted cells. Moreover, we found that inhibition of γ-tubulin sensitized cells to DNA-damaging agents, consistent with its role in DNA repair. Overall, our study characterized γ-tubulin as a new factor in the DNA damage response. This provides an important addition to the nuclear functions of γ-tubulin that have emerged in several recent studies, including its nucleolar association with protein C53, interaction and colocation with PCNA, and partial overlap of the immunofluorescence signal of γ-tubulin with that of RAD51 (Corvaisier and Alvarado-Kristensson, 2020; Corvaisier et al., 2021; Horejsi et al., 2012; Lesca et al., 2005).

Here, we demonstrated that γ-tubulin mediates the mobilization of DNA damage foci, and that its depletion resulted in reduced formation of such foci. These findings parallel our previously reported role of the kinesin motor protein Kif2C in DNA repair (Zhu et al., 2020). Indeed, co-depletion of γ-tubulin and Kif2C confirmed their coordinated action within the same pathway to mobilize DNA damage foci. These new discoveries prompt two intriguing questions. Firstly, how do MT components facilitate the nuclear movement of DNA damage? Secondly, how does MT-mediated mobilization of DNA damage influence the process of DNA repair? These questions underscore the complex interplay between MT dynamics and the DNA damage response.

Although MTs are conventionally regarded as cytoplasmic structures, multiple studies have highlighted their involvement in promoting the mobilization of chromatin following DNA damage (Hauer and Gasser, 2017; Kim, 2022; Lawrimore et al., 2017; Lottersberger et al., 2015; Oshidari et al., 2018; Zhu et al., 2020). Several models have been proposed to elucidate this MT function. For example, cytoplasmic MTs might exert mechanical forces on the nucleus, leading to increased mobilization of nuclear contents, or induce deformations in the nuclear envelope, thereby influencing nuclear organization and movement. Additionally, MTs can coordinate with the linker of nucleoskeleton and cytoskeleton (LINC) complex and nuclear pore complex to guide the mobilization of telomeric and perinuclear chromatin regions (Lottersberger et al., 2015). Intriguingly, MTs have also been implicated in the mobilization of randomly generated DNA damage induced by radiation or etoposide, suggesting an intranuclear involvement of MTs in the DNA damage response (Lottersberger et al., 2015). To this end, our previous studies on Kif2C provide direct evidence supporting the recruitment to sites of DNA damage of this MT component, and the involvement of its MT depolymerase activity in mobilizing DNA DSBs and facilitating foci formation (Zhu et al., 2020). In contrast to the mobilization of damaged DNA, that of a centromere marker or a nuclear RNA processor was not controlled by Kif2C, albeit it was reduced by taxol (Zhu et al., 2020). As such, MTs likely influence chromatin mobilization via distinct mechanisms, including both general stimulation via physical contacts with the nuclear envelope and specific control of damaged DNA via intranuclear MT components. Our present study significantly contributes to the latter model by characterizing γ-tubulin as another MT component directly recruited to DNA damage sites, thereby promoting DNA damage mobilization. The precise coordination between γ-tubulin and Kif2C in this process remains unclear; future investigations are warranted to elucidate the molecular details. For example, it would be interesting to explore whether γ-tubulin and Kif2C mediate the nucleation and depolymerization of α/β-tubulins, respectively, at the DNA damage site.

**Fig. 7.**
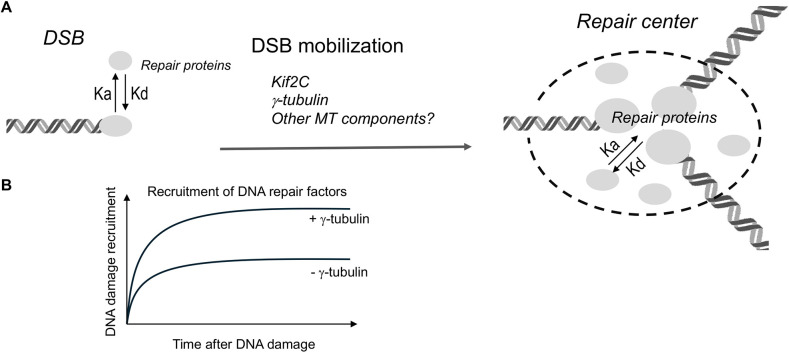
**Microtubule components mobilize DSBs to form repair centers and promote DNA repair.** (A) γ-tubulin, Kif2C and potentially other microtubule (MT) components facilitate DSB mobilization, resulting in the clustering of DSBs and formation of repair centers. An anticipated consequence of the formation of repair centers is increased local concentration of DNA repair proteins and, subsequently, enhanced recruitment of these proteins to DSBs. K_a_, association constant; K_d_, dissociation constant. (B) Mathematical modeling of increased DNA damage recruitment of repair proteins due to γ-tubulin-mediated foci formation. The assumed parameters include cell nucleus size (10 µm), DNA damage focus size (0.5 µm), 5% of repair protein recruitment, 600 total DSBs induced and 15 DSBs clustering within a focus. The overall recruitment of the repair protein to each DSB is projected to be twofold higher with DSB clustering, consistent with our chromatin fractionation analyses.

Mobilization of DNA damage, such as DSBs, can promote their repair. For example, HR and NHEJ depend on homology searching and end relegation, respectively. Both these processes likely involve DSB mobilization. Moreover, our investigations into Kif2C and γ-tubulin have underscored a link between DSB mobilization and the formation of DNA damage foci. From this, we propose that the clustering of DSBs to form foci represents a critical step to ensure efficient DNA repair. Indeed, the formation of DNA damage foci is a hallmark phenomenon of the DNA damage response, and quantitative analyses have revealed that each of these nuclear macrodomains contains numerous DNA breaks. It is conceivable that DSBs in close proximity are mobilized to cluster, and the subsequent formation of repair centers allows for the enrichment of DNA repair proteins to facilitate repair processes (Fig. 7A). Supporting this hypothesis, we demonstrated that depletion of γ-tubulin reduces the chromatin recruitment of both NHEJ and HR proteins. This experimental outcome is well aligned with mathematical modeling that predicted enhanced recruitment of DNA repair factors to DNA damage sites in the presence of γ-tubulin-mediated DNA damage foci formation (Fig. 7B).

Further mechanistic insights are required to substantiate the molecular model proposed in this study. For example, the recruitment of γ-tubulin to laser-induced DNA damage was independent of ATM, ATR or DNA-PK activities, indicating the involvement of a yet-to-be-identified mechanism. γ-tubulin associates with several core factors of NHEJ and HR, but the molecular details and functional significance of these associations remain unclear. Moreover, recent studies have shown that DNA repair factors, such as 53BP1, can exhibit phase separation and liquid-like behavior (Arnould et al., 2023; Kilic et al., 2019). An interesting study in yeast showed that the liquid droplets formed by the HR factor RAD52 fused into DNA repair foci via a mechanism related to intranuclear MT (Oshidari et al., 2020). These findings raise the intriguing possibility that in mammalian cells, nuclear γ-tubulin and MT components similarly facilitate the assembly and movement of DNA repair compartments. This process likely involves complex inter-organelle communications, as evidenced by the role of actin-related protein 2/3 complex in DNA break clustering (Schrank et al., 2018). Finally, although our study is focused on the direct recruitment of γ-tubulin DNA damage and its role in mediating DSB mobility, it is important to also consider the potential influence of γ-tubulin on DNA repair via its established functions in MT and centrosome regulation (Kollman et al., 2011; Mullee and Morrison, 2016).

MT poisons constitute vital components of cancer chemotherapeutics. These drugs target α/β-tubulin to disrupt MT dynamics and are frequently used in combination with radiation or other DNA-damaging drugs. Our study elucidated that suppression of γ-tubulin utilizing two pharmacological inhibitors in conjunction with DNA-damaging agents heightened cellular sensitivity and diminished cell survival. The observed synergistic effect suggests a promising therapeutic strategy for cancer treatment, entailing the combination of γ-tubulin targeting with radiation or DNA-damaging agents. Hence, it would be interesting to explore the targeting of γ-tubulin as an alternative to the clinical utilization of α/β-tubulin inhibitors, potentially to afford more specific effects and ameliorate toxicity. Building on our findings, studies of γ-tubulin targeting using *in vivo* tumor models, and in combination with radiation, chemotherapeutic agents or PARP inhibitors, will be carried out in future studies. Investigations along this path in both preclinical and clinical settings might comprehensively reveal the therapeutic potential of γ-tubulin targeting in cancer therapy.

## MATERIALS AND METHODS

### Cell culture, transfection and treatment

The human cervix carcinoma HeLa cell line was obtained from and authenticated by American Type Culture Collection, and cultured in Dulbecco's modified Eagle medium (DMEM, HyClone) with 10% fetal bovine serum (FBS, HyClone). The head and neck squamous cell carcinoma SCC38 cell line was obtained from and authenticated by the University of Michigan as previously described (Luong et al., 2016). Human bone osteosarcoma epithelial (U2OS) cell lines with or without Kif2C knockout were characterized in our previous study (Zhu et al., 2020). Cells were verified to be contamination free. Transfection of expression vectors was performed using Lipofectamine 2000 transfection reagents (Invitrogen) following the protocol recommended by the manufacturer. mCherry-Gamma-Tubulin-17 was from Addgene (deposited by Michael Davidson; Addgene plasmid #55050; http://n2t.net/addgene:55050; RRID:Addgene_55050). TUBG1_pLX307 was from Addgene (deposited by William Hahn and Sefi Rosenbluh; Addgene plasmid #98376; http://n2t.net/addgene:98376; RRID:Addgene_98376). GFP-tagged DNA polymerase λ was generated by inserting the human DNA polymerase λ gene into pEGFP-C1 vector (Clontech). *TUBG1* siRNA (#1, hs.Ri.TUBG1.13.1; #2, hs.Ri.TUBG1.13.2; Integrated DNA Technologies) or a control non-targeting siRNA (51-01-14, Integrated DNA Technologies) was transfected using Lipofectamine RNAiMAX (Invitrogen). The The KUBTEC Scientific XCELL 50 benchtop X–ray irradiator system was used to induce DNA damage in cells. An X-ray dose of 20 Gy, delivered using this low power (10–50 kV, 1.5 Gy/min) irradiator, was selected because it effectively induced DSBs, as evidenced by γ-H2AX signaling, with the majority of the DSBs being repaired within a few hours.

### Chromatin fractionation assay

Cells were detached using trypsinization and suspended in DMEM. The cell pellet was washed with PBS and centrifuged. The pellet was then partitioned for subsequent analyses. For the whole cell lysates, the pellet was processed with SDS. The remaining pellet was subjected to chromatin fractionation for the isolation of the chromatin-bound fraction. The chromatin fractionation involved resuspending the pellet in 100 µl of buffer A [10 mM HEPES, pH 7.9, 10 mM KCl, 1.5 mM MgCl_2_, 0.34 M sucrose, 10% glycerol, 1 mM dithiothreitol (DTT), 0.1% Triton X-100 and protease inhibitors]. This suspension was incubated on ice for 8 min. The pellet obtained was then resuspended in 100 µl of buffer B (3 mM EDTA, 0.2 mM EGTA, 1 mM DTT and protease inhibitors) and incubated at room temperature for 30 min to pellet the chromatin. The chromatin pellet was further washed with buffer B and resuspended in SDS sample buffer to constitute the chromatin fraction.

### DNA repair assays

Chromosome-integrated, I-SceI-induced HR or NHEJ assays were performed as in our previous study (Zhu et al., 2020). The reporter cells HeLa-Dr or U2OS-EJ5, as in Zhu et al. (2020), were transfected with control or γ-tubulin siRNA. I-SceI endonuclease was expressed using a lentiviral vector [pCVL SFFV-EF1s HA.NLS.Sce(opt); deposited by Andrew Scharenberg, Addgene plasmid #31479, RRID:Addgene_31479]. The expression of HA–I-SceI was confirmed by immunoblotting, as in Zhu et al. (2020). Following a 48-h incubation, DNA repair was measured by immunoblotting to assess GFP expression in relation to SMC1 expression.

### Immunoblotting and chemicals

SDS-PAGE and immunoblotting were carried out using the following antibodies: anti-artemis (D7O8V, #13381, 1:500), anti-Ku80 (C48E7, #2180, 1:1000), anti-RAD51 (D4B10, #8875, 1:800), anti-DNA-PKcs (E6U3A, #38168, 1:1000), anti-Ku70 (D10A7, #4588, 1:1000), anti-RPA70 (#2267, 1:1000), anti-DNA ligase IV (D5N5N, #14649, 1:1000), anti pATM/ATR substrate (#6966, 1:1000) and anti-XLF (#2854, 1:1000) from Cell Signaling Technology (Beverly, MA, USA); anti-α-tubulin (ab7291, 1:1000) and anti-γ-tubulin (ab11317, 1:1000) from Abcam (Cambridge, MA, USA); anti-γ-H2AX (Ser 139) (sc-517348, 1:1000), anti-total H2AX (sc-517336, 1:1000), anti-PARP1 (B-10, sc-74470, 1:1000), anti-histone H3 (1G1, sc-517576, 1:1000), anti-DNA polymerase λ (E-11, sc-373844, 1:1000), anti-RPA32 (9H8, sc-56770, 1:1000) and anti-RPA70 (H-7, sc-48425, 1:1000) from Santa Cruz Biotechnology (Dallas, TX, USA).

The chemicals used in this study include: gatastatin G2 (γ-tubulin inhibitor) (FNK-FDV-0040) from Diagnocine (Tokyo, Japan); MR-3 (S3888), NU7441 (S2638), and bleomycin (S1214) from Selleckchem (Houston, TX, USA); etoposide (341205), caffeine (C0750) and doxorubicin (D1515) from Sigma-Aldrich (St. Louis, MO, USA); and hydroxyurea (Q9994) from MP Biomedicals (Solon, OH, USA).

### Immunofluorescence and imaging

Immunofluorescence and imaging were performed as in our previous studies (Li et al., 2023; Yamamoto et al., 2014). Cells were grown in a glass-bottomed dish, washed with PBS twice, and fixed with 3% formaldehyde with 0.1% Triton X-100 for 30 min. 0.05% saponin in PBS was used to permeabilize the fixed cells, followed by blocking with 5% goat serum for 30 min. Primary antibodies were diluted in blocking buffer and incubated with the cells for 2 h. The following primary antibodies were used: anti-γ-tubulin (ab11317, 1:200) and anti-poly (ADP-Ribose) polymer (ab14459) from Abcam (Cambridge, MA, USA), anti-p-ATM (Ser1981) (200-301-400, 1:200) from Rockland (Pottstown, PA, USA), and anti-53BP1 (#4937, 1:500) from Cell Signaling Technology (Beverly, MA, USA). The cells were then incubated with Alexa Fluor-conjugated secondary antibodies [goat anti-rabbit IgG (H+L) cross-adsorbed secondary antibody, Alexa Fluor 555; Thermo Fisher Scientific; 1:2000] for 1 h at room temperature. The nuclei of cells were stained with 4′,6-diamidino-2-phenylindole (DAPI), and the stained cells were imaged using a Zeiss Axiovert 200M inverted fluorescence microscope. Laser micro-irradiation was performed using a 405 nm laser under the Zeiss Axiovert 200M microscope with Marianas Software (Intelligent Imaging Innovations, Denver, CO, USA). Cells were not pre-sensitized using this micro-irradiation system. Additionally, we also performed laser micro-irradiation with pre-sensitization. Here, confocal microscopy for high-resolution laser micro-irradiation imaging was performed using a Zeiss 880 confocal laser scanning microscope, and laser micro-irradiation was carried out using a 405 nm laser. 1 h prior to laser micro-irradiation, cells were pre-treated with Hoechst 33342 (Invitrogen) at 1 μg/ml.

### Immunoprecipitation

Anti-γ-tubulin antibody (sc-51715, 1:200) from Santa Cruz Biotechnology or anti-Ku80 antibody (D10A7, #4588, 1:1000) from Cell Signaling Technology was conjugated with magnetic beads (Thermo Fisher Scientific) and incubated with HeLa cell lysates. The beads were re-isolated using a magnet separator, washed three times with PBS, and then analyzed using SDS-PAGE. The input, control immunoprecipitation products using blank beads, and γ-tubulin or Ku80 immunoprecipitation products were analyzed by immunoblotting. The control and anti-γ-tubulin immunoprecipitation products, performed with or without bleomycin treatment (20 µM, 3 h), were also subjected to proteomic analysis at the proteomic core facility of the University of Nebraska Medical Center.

### Microscopic analysis of DNA damage foci formation and mobility

The Apple-53BP1trunc plasmid (encoding 53BP1 amino acids 1220–1711) was deposited by Ralph Weissleder to Addgene (plasmid #69531; RRID:Addgene_69531) (Yang et al., 2015). EGFP–53BP1 was expressed through transient transfection of a plasmid DNA vector, as described in our previous study (Zhu et al., 2020). Image acquisition was carried out using a Zeiss spinning disk confocal microscopy system equipped with a 63× oil Plan Apochromat oil objective. Foci analysis and tracking were performed using FIJI (ImageJ 2) (National Institutes of Health), and the foci number was quantified using the ‘analyze particles’ counting function. Data analysis and graph presentations were performed using GraphPad Prism 9 (GraphPad Software, La Jolla, CA, USA) and Microsoft Excel. For foci mobility, time-lapse recordings were done with images acquired every 30 s for 10 min. Data analysis and graph presentations were performed using Microsoft Excel and KaleidaGraph (Synergy). Two-tailed unpaired Student's *t*-test was used for statistical analysis. Mean square displacement was calculated as previously described (Zhu et al., 2020).

### Cell viability assay

HeLa and SCC38 cells were seeded into 96-well transparent plates at 10–30% confluency. After overnight incubation, the cells were treated with or without gatastatin G2, MR-3, etoposide and doxorubicin at the desired concentrations. On the day of analysis, DMEM was replaced by WST-8 solution from the Cell Counting Kit 8 (CCK-8) (Abcam, ab228554) and the plates were incubated for 1 h. Thereafter, the increase in absorbance was determined using a BioTek ELx808 spectrophotometer at 450 nm as per the instructions of the manufacturers for colorimetric analyses.

### Spheroid cell analysis

SCC38 cells were plated onto Nunclon Sphera 24-well plates (Thermo Fisher Scientific), and maintained in DMEM supplemented with 10% FBS. Cells were exposed to varying concentrations of gatastatin G2, MR-3, etoposide and doxorubicin as per the experimental requirements. The response to the treatments was assessed by measuring the diameters of the formed spheroids. Data were gathered from three independent experiments, and statistical analyses were performed using one-way ANOVA with Dunnett's multiple post hoc test.

### Statistical analysis

All data are represented as the mean±s.e.m. or mean±s.d. of three to five independent experiments. The data were analyzed using GraphPad Prism 9. Two-tailed unpaired Student's *t*-tests, one-way ANOVA with Dunnett's multiple post hoc test and two-way ANOVA with Tukey's multiple post hoc test were performed to determine the statistical significance for paired samples. *P<0.05, **P<0.01 and ***P<0.001 were considered significant.

## Supplementary Material



10.1242/joces.262255_sup1 Supplementary information
