# Supplementary material for: γ-tubulin mediates DNA double-strand break repair
Source: J Cell Sci. 2025 Mar 26;138(6):jcs262255. doi: 10.1242/jcs.262255 (PMC12050090; doi:10.1242/jcs.262255)
Supplement: Supplementary information [file joces-138-262255-s1.pdf]

A

	exp 1	exp 2	exp 3	control
γ-tubulin	6	7	6	0
α-tubulin	28	26	43	8
β-tubulin	25	38	33	10

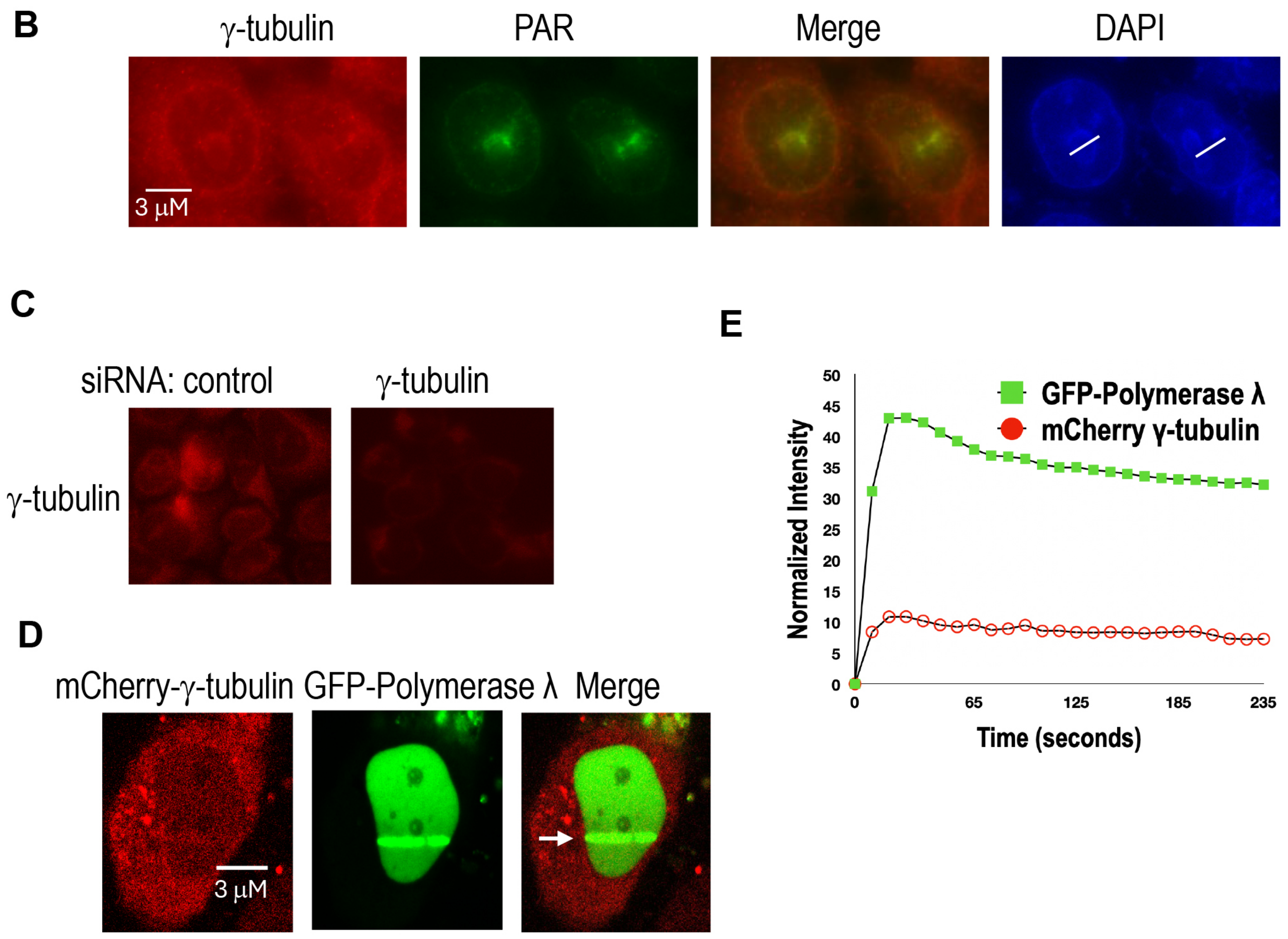

Fig. S1. γ- tubulin is recruited to DNA damage sites. (**A**) As described in our previous study (Zhu et al., 2020), a pull-down experiment was performed in *Xenopus* egg extracts, using biotin dA-dT (70 mer) conjugated on streptavidin magnetic beads (New England Biolabs). The pull-down products, and control pull-down using empty beads, were analyzed by mass spectrometry. Several tubulins were recovered in the pull down, as shown with the numbers of peptides recovered. (**B**) HeLa cells were laser-microirradiated and analyzed by immunofluorescence (IF) for **γ**-tubulin and poly(ADP-ribose) (PAR). The images were taken 3 min post laser-treatment. The region of laser micro-irradiation is denoted by the white lines. (**C**) HeLa cells treated with control or γ-tubulin siRNA were analyzed by IF for γ-tubulin. (**D**) HeLa cells expressing mCherry-γ-tubulin and GFP-Polymerase λ were laser micro-irradiated and imaged. This laser system involved pre-sensitization and confocal imaging, as in Fig. 1D. (**E**) Cells were imaged as in panel D, the recruitment kinetics of mCherry-γ-tubulin and GFP- Polymerase λ are shown.

Journal of Cell Science • Supplementary information

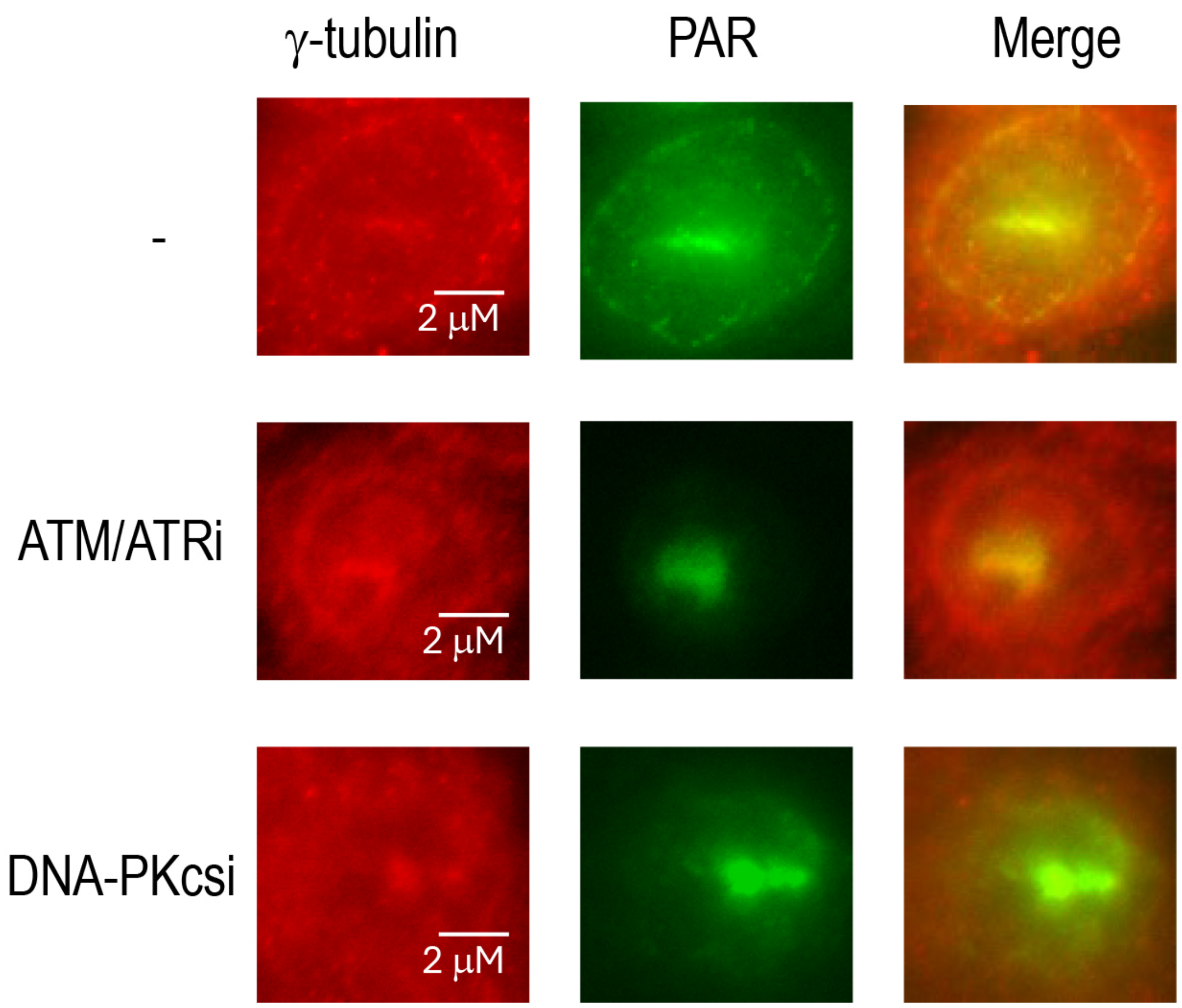

Fig. S2. γ- tubulin recruitment to DNA damage sites is independent of ATM, ATR and DNA-PKcs activities. HeLa cells were treated with laser micro-irradiation and analyzed by IF, as in Fig. S1B. Cells were pre-treated with or without ATM/ATR inhibitor (caffeine, 4mM) or DNA-PKcs inhibitor (NU7441, 20 uM), as indicated.

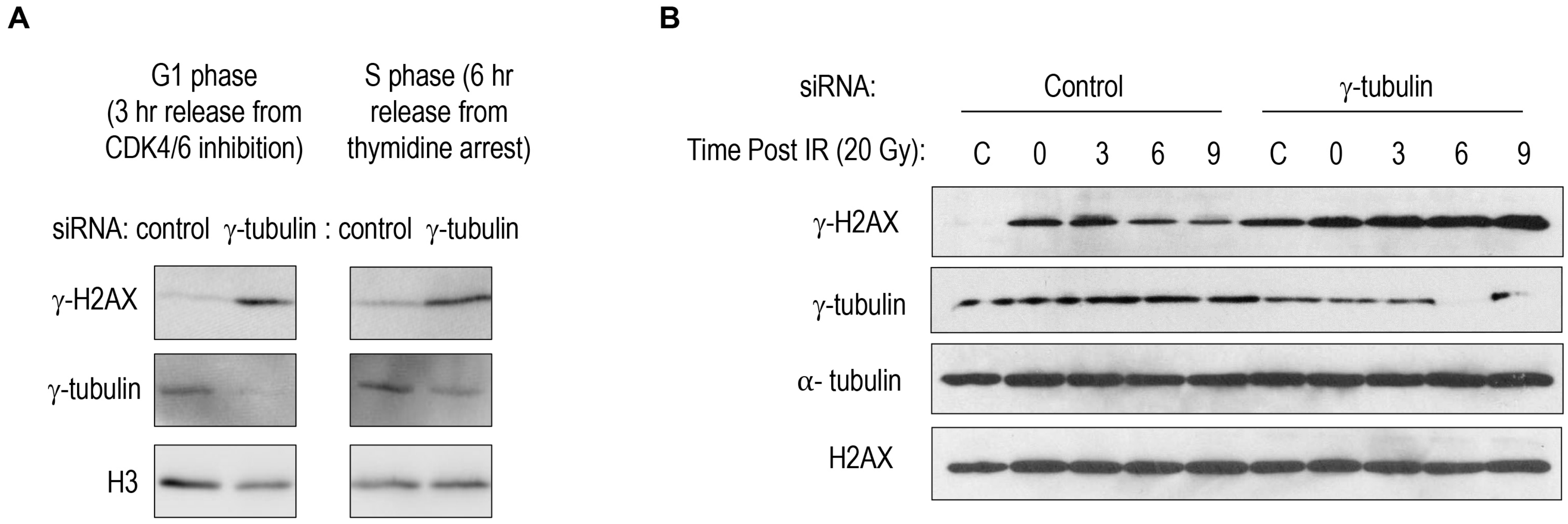

Fig. S3. The role of γ-tubulin in DNA repair. (**A**) Inhibition of γ-tubulin in interphases leads to DNA damage accumulation. Cells treated with control or γ-tubulin siRNA were synchronized in G1 or S phases, as indicated, and analyzed by IB for the indicated proteins. **(B)** γ-tubulin depletion delays DNA repair. HeLa cells with control or γ-tubulin siRNA were treated with 20 Gy IR, as described in Materials and Methods, followed by repair/recovery for 0, 3, 6, and 9 hours as indicated. The samples were analyzed by IB.

Journal of Cell Science • Supplementary information

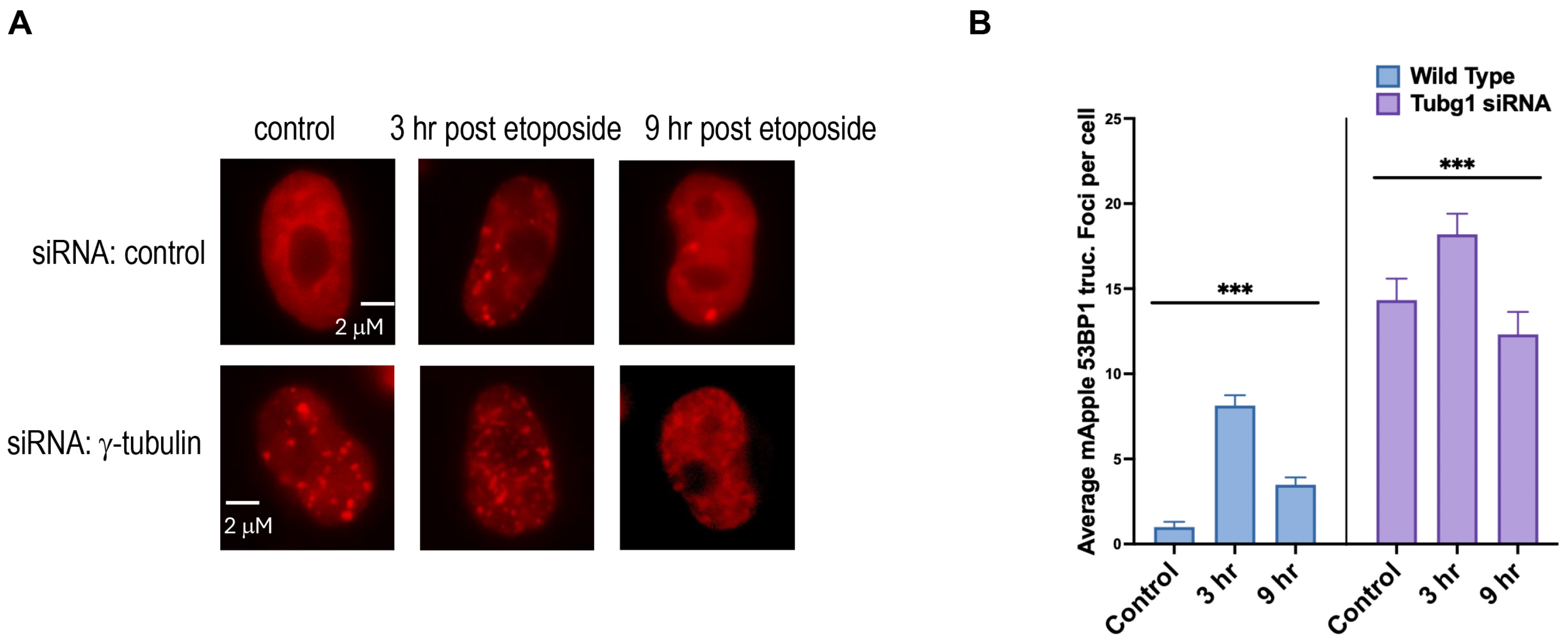

Fig. S4. γ-tubulin depletion delays DNA repair. (A, B) Hela cells with control or γ-tubulin siRNA, were transfected with mApple-53BP1-truc, as described in Material and Methods. Cells were treated with 1 μM etoposide for 1 hour, followed by repair/recovery for 0, 3 and 9 hours as indicated. Representative images are shown in panel A. Quantification is shown in panel B. The average number of mApple-53BP1-truc foci was counted in >20 cells for each condition and analyzed using ANOVA (***$p<0.001$).

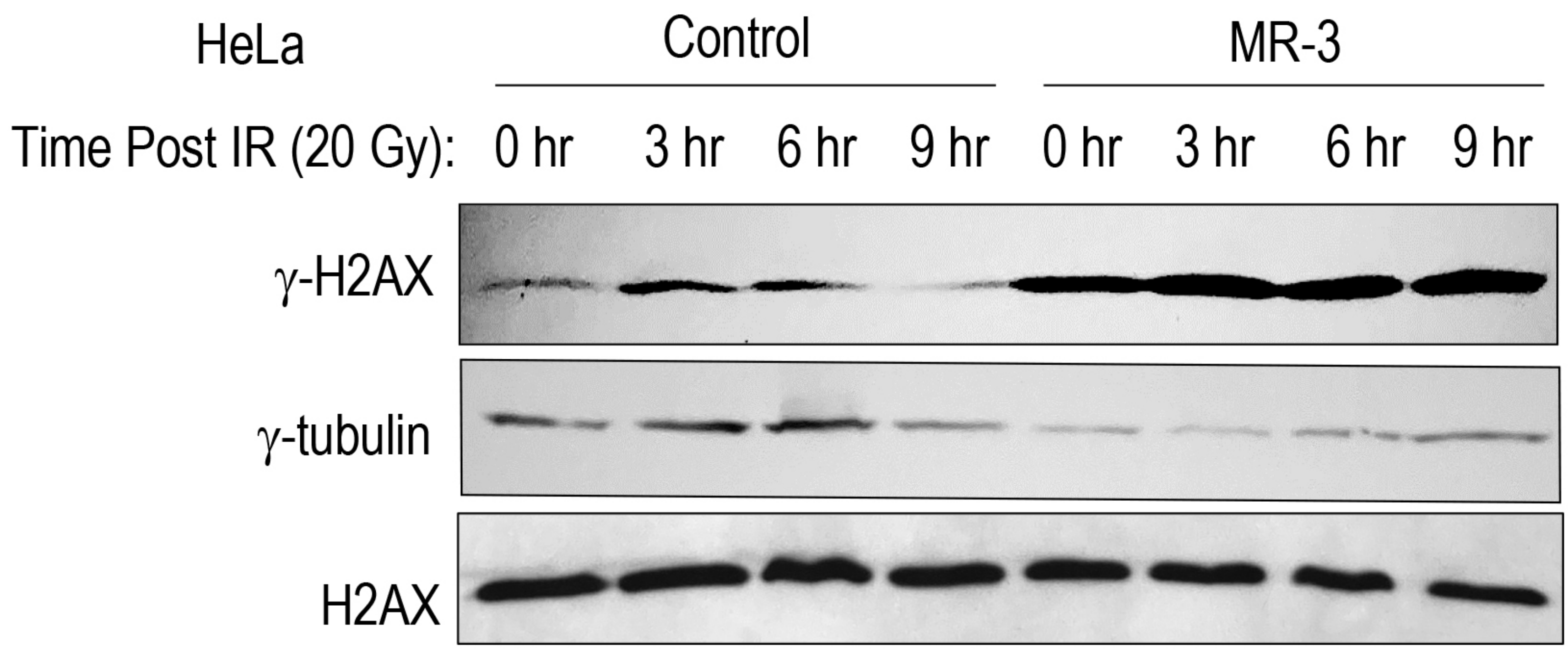

Fig. S5. γ-tubulin inhibition delays DNA repair. HeLa cells were treated with 15 μM MR-3, followed by repair/recovery for 0, 3, 6, and 9 hours as indicated. The samples were analyzed by IB for the indicated proteins.

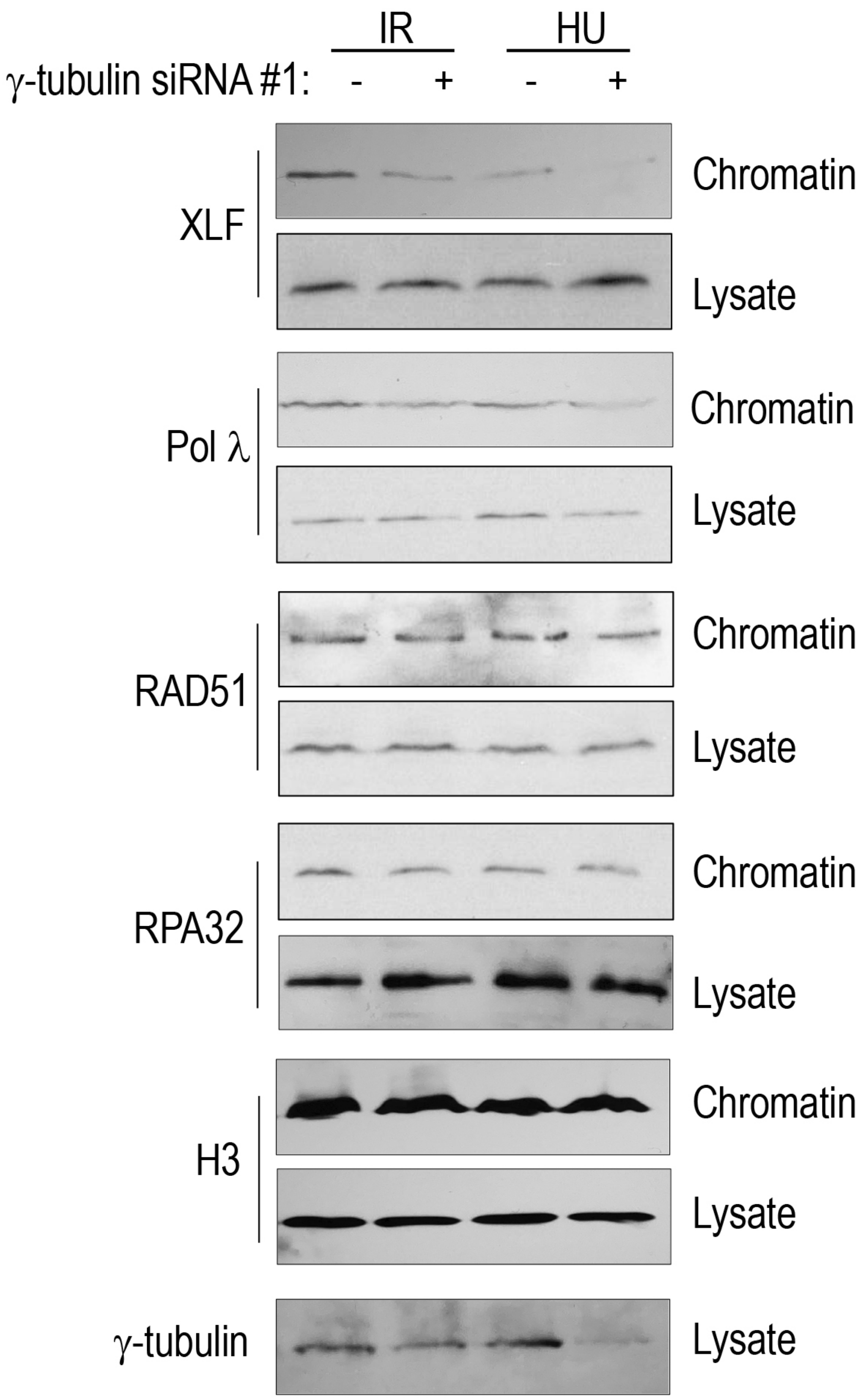

Fig. S6. γ-tubulin depletion reduced the chromatin recruitment of DSB repair factors. HeLa cells treated/untreated with γ tubulin siRNA (24 hr) were either irradiated with 20 Gy of X-ray, or treated with Hydroxyurea, followed by 3 hr incubation. Chromatin fractionation was performed as described in Material and Methods. The lysates and chromatin samples were analyzed by IB.

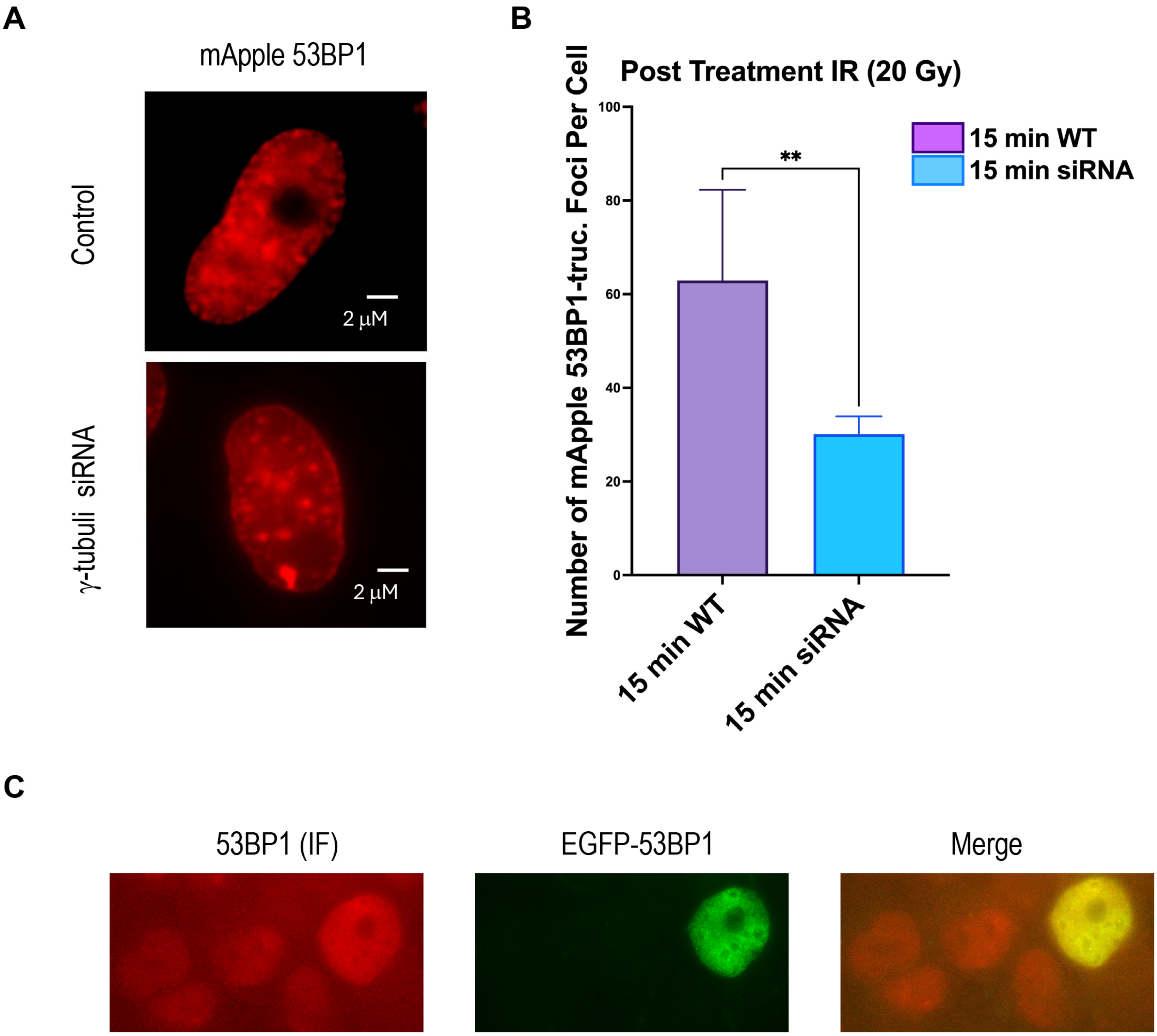

Fig. S7. γ-tubulin depletion attenuates foci formation. **(A, B)** HeLa cells treated/untreated with γ tubulin siRNA (24 hr) were irradiated with 20 Gy of X-ray, followed by 15 min incubation. The cells were fixed with 3% formaldehyde with 0.1% Triton X-100 for 30 min. Representative images are shown in panel A, and quantification of 53BP1 foci is shown in panel B (N>100). All data were collected from at least three independent experimental sets as mean ± S.D.; significant: **p<0.01 by unpaired 2-tailed Student's t-test. **(C)** The expression level of EGFP-53BP1. HeLa cells transfected with EGFP-53BP1 were analyzed by IF for 53BP1 to indicate the levels of endogenous and overexpressed 53BP1.

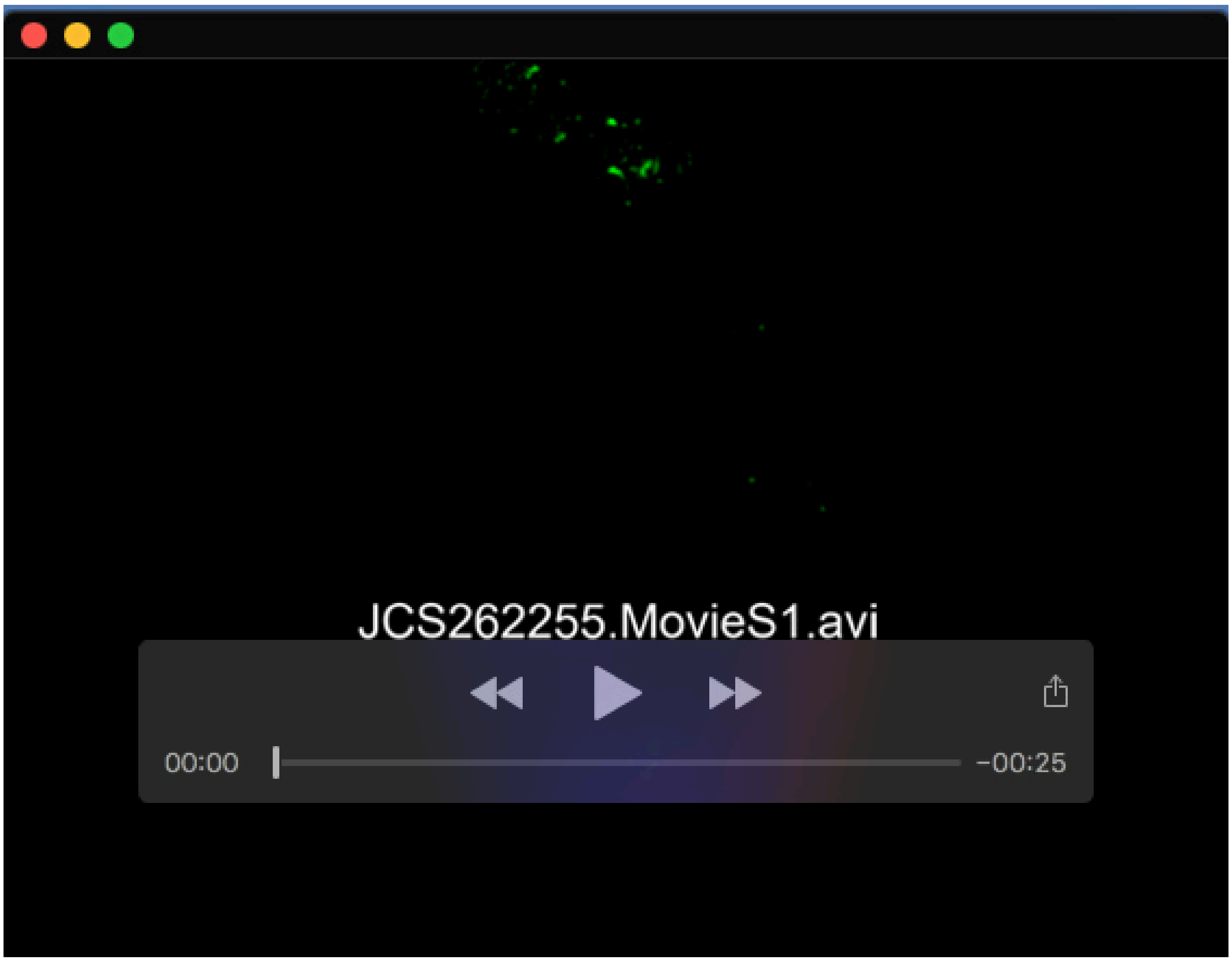

Movie 1. DNA damage (etoposide)-induced GFP-53BP1 foci.

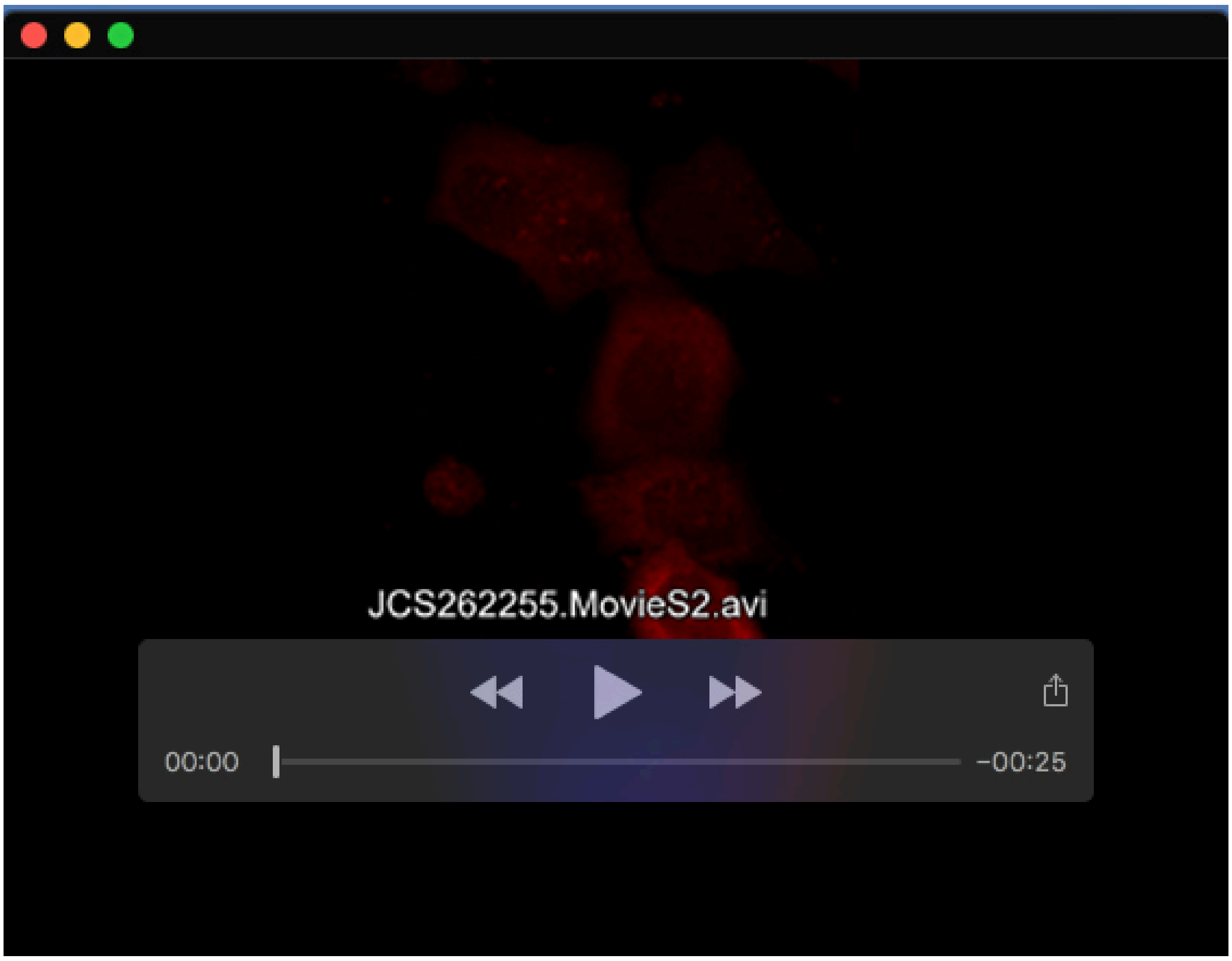

Movie 2. DNA damage (etoposide)-induced mCherry-γ-tubulin foci.

Journal of Cell Science • Supplementary information

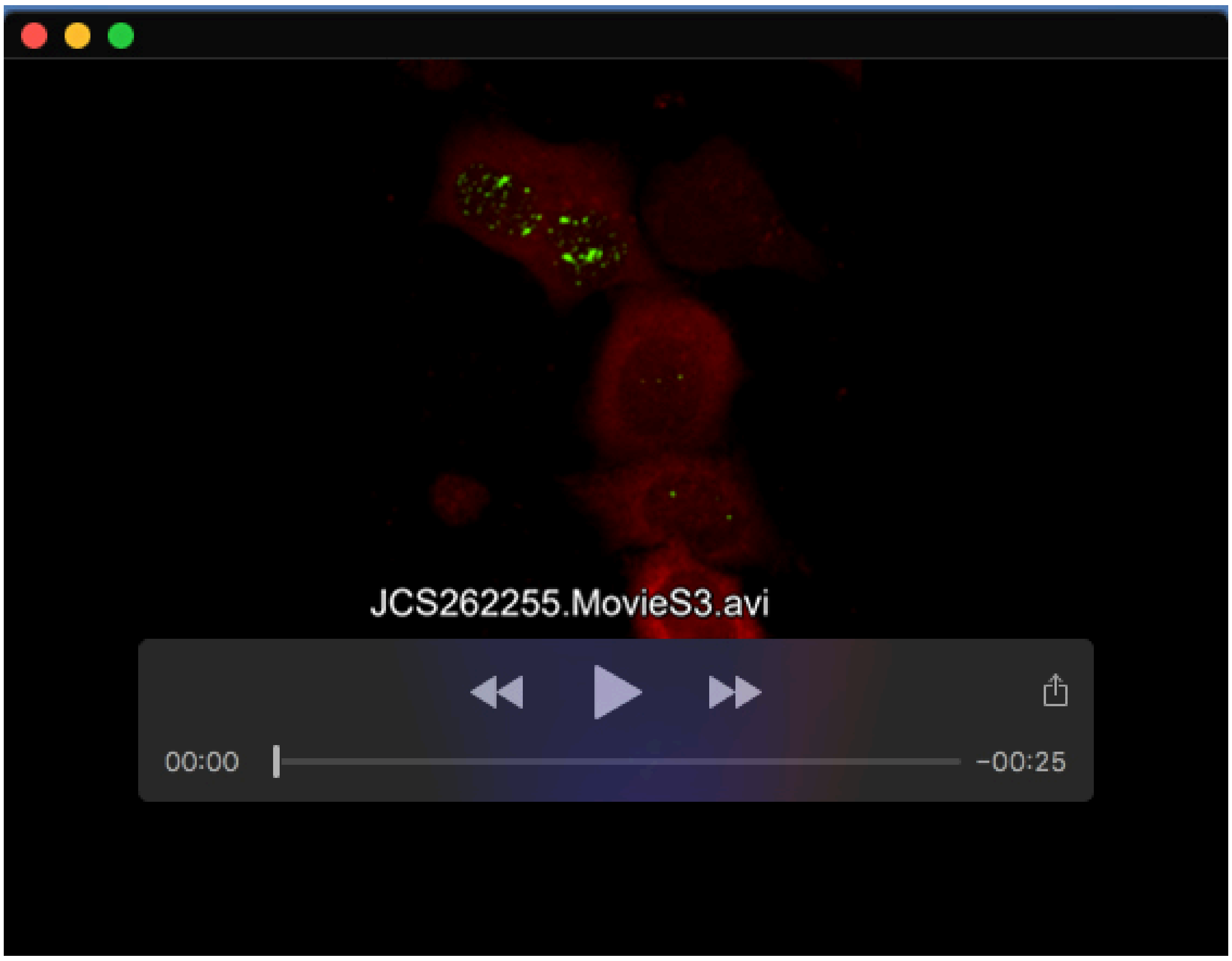

Movie 3. Merge of GFP-53BP1 and mCherry-γ-tubulin foci.

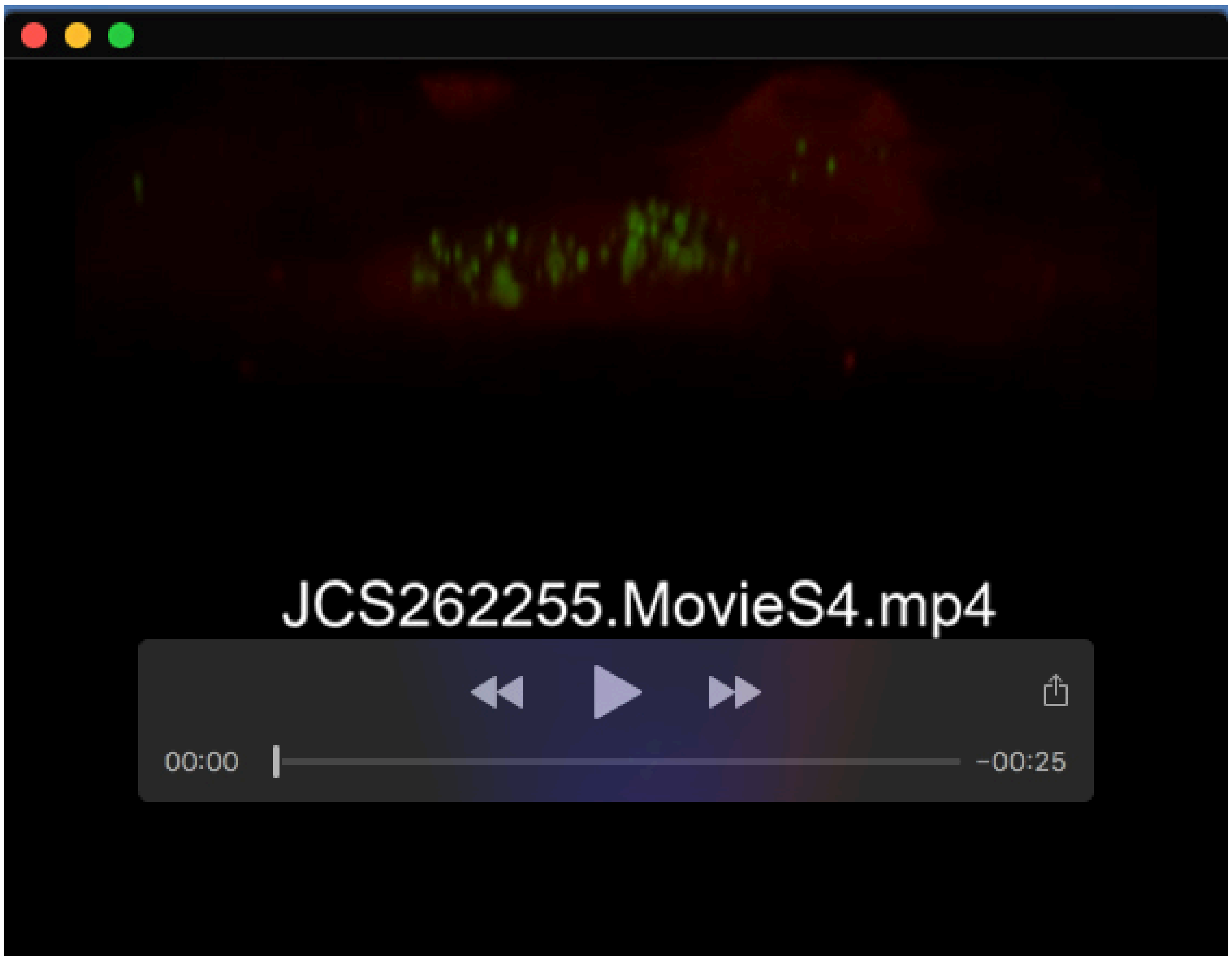

Movie 4. Merge of GFP-53BP1 and mCherry-γ-tubulin foci (rotation).

Reference

Zhu, S., M. Paydar, F. Wang, Y. Li, L. Wang, B. Barrette, T. Bessho, B.H. Kwok, and A. Peng. 2020. Kinesin Kif2C in regulation of DNA double strand break dynamics and repair. *eLife*. 9.

Journal of Cell Science • Supplementary information
