# Supplementary material for: γ-tubulin mediates DNA double-strand break repair
Source: J Cell Sci. 2025 Mar 26;138(6):jcs262255. doi: 10.1242/jcs.262255 (PMC12050090; doi:10.1242/jcs.262255)
Supplement: Supplementary file 1 [file joces-138-262255_review_history.pdf]

Journal of Cell Science

γ-tubulin mediates DNA double-strand break repair

Abhishikt David Solomon, Odjo G. Gouttia, Ling Wang, Songli Zhu, Feifei Wang, Yanqiu Li, Mohammadjavad Paydar, Tadayoshi Bessho, Benjamin H. Kwok and Aimin Peng
DOI: 10.1242/jcs.262255

Editor: Megan King

Review timeline
Original submission: 4 May 2024
Editorial decision: 24 June 2024
First revision received: 25 November 2024
Accepted: 20 December 2024

Original submission

First decision letter

MS ID#: JOCES/2024/262255

MS TITLE: Gamma-tubulin Mediates DNA Double Strand Break Repair

AUTHORS: Abhishikt David Solomon, Ling Wang, Songli L Zhu, Feifei Wang, Yanqiu Li, Mohammadjavad Paydar, Tadayoshi L Bessho, Benjamin Kwok, and AIMIN PENG

We have now reached a decision on the above manuscript.

To see the reviewers' reports and a copy of this decision letter, please go to: https://submit-jcs.biologists.org and click on the 'Manuscripts with Decisions' queue in the Author Area. (Corresponding author only has access to reviews.)

As you will see, the reviewers raise a number of substantial criticisms that prevent me from accepting the paper at this stage. They suggest, however, that a revised version might prove acceptable, if you can address their concerns. If you think that you can deal satisfactorily with the criticisms on revision, I would be pleased to see a revised manuscript. We would then return it to the reviewers.

Please ensure that you clearly highlight all changes made in the revised manuscript. Please avoid using 'Tracked changes' in Word files as these are lost in PDF conversion.

I should be grateful if you would also provide a point-by-point response detailing how you have dealt with the points raised by the reviewers in the 'Response to Reviewers' box. Please attend to all of the reviewers' comments. If you do not agree with any of their criticisms or suggestions please explain clearly why this is so.

Reviewer 1

Advance summary and potential significance to field

The manuscript demonstrates the role of γ-tubulin during the repair of double strand breaks (DSBs) in human cells. The topic is very timely and extremely interesting. The authors clearly show that a fraction of γ-tubulin is recruited to DNA damage in the nucleus and colocalize with several repair

© 2025. Published by The Company of Biologists under the terms of the Creative Commons Attribution License (https://creativecommons.org/licenses/by/4.0/).

factors such as PAR, ATM, 53BP1 and Polymerase l. Although the precise function of γ-tubulin during DNA repair is not totally elucidated, they show that depletion of γ -tubulin impedes DNA repair both by homologous recombination and non-homologous recombination. In addition, γ-tubulin is implicated in the higher mobility of 53BP1 repair foci, which provokes the clustering of 53BP1 focus. Considering the important role of γ-tubulin, they finally show that inhibitors of γ-tubulin enhance the cytotoxic effect of DNA damaging agents.

The manuscript is very well written, clear and concise. The results clearly indicate the recruitment of γ-tubulin at DNA lesions and its importance for DNA repair. Some results need to be clarified. For example, it would important to show the kinetics of γ-tubulin recruitment at DNA lesions, the liquid properties of 53BP1 foci and the way they interact with γ-tubulin could be discussed, the results on Rad51 are not very convincing, replicates of IP experiments should be shown, mean square displacements curves of 53BP1 should be shown and fitted.

In short, the manuscript is very interesting, but certain results need some clarifications.

Comments for the author

The manuscript is very interesting but several points should be clarified and discussed.

To induce DNA damage on cells, the authors used 405 nm laser micro-irradiation under a Zeiss Axiovert 200M Microscope. Do they pre-sensitize the cells before irradiation? If they don't pre sensitize the cells, what kind of DNA damages are induced with the 405 nm laser? What is the laser power at the sample in W per cm2?
After micro-irradiation, they observe a colocalization of γ-tubulin with PAR and other repair factors. The time after micro-irradiation at which the IF was performed needs to be indicated. It is known that PAR is recruited just a few seconds after damage, and is disassembled 10 minutes after damage. The data in Figure 2C suggests that γ-tubulin is recruited very quickly after irradiation. It would very informative to perform a timelapse of the γ-tubulin recruitment after micro-irradiation in live cells.

Figure 2, is it possible to quantify the immune-precipitation bands ?
Figure 2B, for PARP1 and especially for Rad51, there is a clear band in the absence of bleomycin. The authors mention that there is "a notable level of γ-tubulin-Rad51" association, albeit accompanied by a lower non specific signal".
Is it simply an artefact or is there non-specific interaction of Rad51 and PARP1 with γ-tubulin in the absence of damage?

In figure 4, the authors use I-SceI-induced HR or NHEJ reporter cells. Can they precise the timing and the efficiency of the I-SceI cutting in these cell lines
?
Figure 5, the authors measure the mobility of 53BP1 foci in living cells. For that, they use a U2OS cell line expressing EGFP-53BP. However, there is no mention of this cell line in the material and method. Is it an endogenous fusion of EGFP to 53BP1? is it a stable cell line expressing EGFP-53BP1, or is it a transitory transfection of a plasmid? Several studies showed that 53BP1 proteins form membrane less condensates via liquid liquid phase separation (doi: 10.15252/embj.2018101379; https://doi.org/10.1038/s41586-023-06635-y).
Changing the physiological level of proteins expression might change the condensate properties. It is thus, essential to favor experiments using endogenous tagged proteins or to carefully check the level of expression by Western Blot.
In figure 5A, the authors analyze the mobility of 53BP1 foci: can they show a typical trajectory in WT versus siRNA treated cell ? Can they show the mean square displacement and fit the curve to extract diffusive parameters ?
When the author use an Apple-53BP1trunc plasmid, can they explain what it is 53BP1trunc and is it overexpressed ?

Discussion:

© 2025. Published by The Company of Biologists under the terms of the Creative Commons Attribution License (https://creativecommons.org/licenses/by/4.0/).

In the discussion, the physical nature of 53BP1 should be discussed. Do foci look spherical or not? in Oshidari 2020, Rad52 droplets exhibit wetting behavior against MT. Do they see this phenomenon here for 53BP1 against tubulin?
The role of AR2/3 should be mentioned as well.

Minor comments:
Replicates of IP and IB should be shown in supplementary data.
In the material and methods, they authors need to provide de references of all their anti-bodies against g-tubulin, ATP, PAR, 53BP1 polymerase l. Have they tried anti bodies against Rad51 and if yes, what do they see?

When they use bleomycin of X rays, can they give an idea of the type of damage and the number of breaks induced? For example, 20 Gy of X rays seems very high for human cells.

Reviewer 2

Advance summary and potential significance to field

The authors present data suggesting a nuclear role for gamma-tubulin in DNA double-strand break (DSB) repair in human cells. The data potentially suggest that gamma-tubulin is recruited to DNA damage sites, promotes the targeting of repair proteins (NHEJ and HR factors) to the chromatin fraction of cells exposed to DNA damage, and mediates repair by NHEJ and HR. The authors observed that gamma-tubulin is required for DSB mobilization and potentially works in the same pathway as KIFC2, a microtubule-depolymerizing kinesin. A tentative mechanism is proposed where somehow nuclear gamma-tubulin works in concert with KIFC2 to mediate DSB mobilization and clustering, which would, in turn, somehow increase the local concentration of repair factors, thereby increasing accurate repair and cell survival. The authors also suggested a potential therapeutic avenue of further sensitizing cancer cells to irradiation or chemotherapeutics by pharmacologically inhibiting gamma-tubulin. The authors do acknowledge late in the discussion that various tubulin isoforms and microtubules could promote repair by operating via different mechanisms, including through the cytoskeleton exerting forces onto chromatin by pushing onto the nuclear envelope.

Overall, investigating the role of tubulin in DNA double-strand break repair and mobility is a topical area in the DNA repair field and this study may demonstrate that nuclear gamma-tubulin is involved in human DNA repair. Although the study is of potential interest, major revisions are necessary.

Comments for the author

Major comments:

*Mechanistically, the study does not provide a clear and well-supported mechanism through which gamma-tubulin promotes DSB repair. Although gamma-tubulin appears to potentially impact DSB mobility and the recruitment of NHEJ and HR factors to chromatin, it is unclear how these processes are occurring. The authors should consider the following questions:
-How is the entire process triggered upon DSB induction? For example, do the DNA Damage Response kinases ATM, ATR, and/or DNAPK increase the nuclear localization and/or chromatin association of gamma-Tub?
-How might gamma-tubulin promote the recruitment of both NHEJ and HR factors?
-Does gamma-tubulin interact with KIF2C? Is gamma-tubulin required for KIF2C-mediated DSB repair?
-Is the role of gamma-tubulin related to or distinct from the role of alpha/beta-Tubulin? considering the better-documented roles of alpha/beta-tubulin in DNA repair, it is also important to assess whether the disruption of gamma-tubulin disrupts alpha/beta-tubulin-dependent DSB repair as well.
-Clustering is not generally viewed as a repair-promoting mechanism. Rather, it is a process primarily affecting hard-to-repair or persistent breaks. So, it is very unlikely that a potential increase in DSB clustering would be a primary mechanism to drive physiologically relevant DNA repair events.

© 2025. Published by The Company of Biologists under the terms of the Creative Commons Attribution License (https://creativecommons.org/licenses/by/4.0/).

-Previous studies have provided evidence that tubulin/microtubules mediate DNA repair in different organisms via various nuclear and cytoplasmic mechanisms, which are generally mentioned although not accurately discussed or cited in the introduction and discussion sections. The authors should better explain how their proposed mechanism intersects with these and other studies in the literature. Especially, the mechanism proposed here, although still not supported by the data, seems illogical. How can a mechanism promoting DSB clustering as a primary function serve to maintain accurate DSB repair and preserve genome stability and viability in the long term?

*Is the herein-studied role of nuclear gamma-tubulin distinct from its role in nucleating microtubules at the centrosome and other MTOCs? Does nuclear gamma-tubulin nucleate or prevent the nucleation of short microtubules in the nucleus upon DNA damage induction? How is this intersecting with the role of KIF2C as a microtubule-depolymerizing kinesin? Moreover, the centrosome can harbor various DNA repair proteins (see review Mullee and Morrison, 2015). Is it possible that the mechanism suggested in this manuscript relates to changes in the centrosome upon DNA damage?

*A substantial proportion of the data provided in this study is not of a high-quality, leaving this reviewer with the impression that many claims are not supported by the data presented. There are a number of issues regarding the imaging data provided:
-In Figure 1, there is a lot of background in the images, especially in the gamma-tubulin red channel. Gamma-tubulin is typically enriched in the centrosome with minimal cytoplasmic staining. The images provided raise questions about the quality and specificity of the antibodies/imaging. An inverted fluorescence microscope was used to capture the images used in this study. A confocal microscope and 3D images might provide higher-resolution 3D. Additionally, knockdown controls assessing whether the weak nuclear gamma-Tubulin signals reported are indeed abolished upon gamma-Tubulin knockdown are missing and sorely needed.
-In Figure 1C, it appears that the left cell was irradiated along the diameter of the nucleolus, resulting in a strange pattern in PARP recruitment (i.e., at the periphery of the nucleolus). Is that the case? Please clarify either way.
-None of the imaging figures are accompanied by quantitative data. For example, colocalization can be accompanied by intensity profile graphs. Moreover, the gamma-tubulin ‘foci’ depicted in Figure 1D should be accompanied by quantifications that compare control and DNA damage conditions on a single-cell and cell population level.
-In Figure 1E, it is difficult to see the recruitment of mCherry gamma-tubulin to the sites of damage. In fact, these data suggest that the protein is not recruited to the site of damage, raising doubt about the overall model presented.
-There are no scale bars on any of the images provided.

*In Figure 1, what is occurring to gamma-tubulin upon DSB induction? Specifically, the authors should show whether the levels of gamma-tubulin change overall within the same experimental conditions (e.g., with a western blot) and whether the nuclear vs. cytoplasmic localization of gamma-tubulin changes via a nuclear fractionation. Additionally, it appears that the levels of gamma-tubulin do change upon DNA damage in certain figures (e.g., Figure 4C and first two lanes) but do not change in other figures (e.g., Figure 4D). How might the authors explain this discrepancy?

*The authors claim a substantial role for gamma-tubulin in promoting the recruitment of key DSB repair proteins (NHEJ and HR) to chromatin. To this end, a series of chromatin fractionations are shown (Figure 4), but the data are not very convincing. For example, in Figure 4F, there is only a very slight reduction in Rad51’s recruitment to chromatin following gamma-tubulin knockdown, despite gamma-tubulin disruption strongly decreasing the repair of HR and NHEJ reporters. In fact, the overall data assessing links between gamma-tubulin and HR repair is weak. Specifically, no HR-related protein was shown in the dsDNA IP (Figure 1A) as a positive control and no HR protein appeared in the proteomic analysis (Figure 2A); the blot for Rad51 in the gamma-tubulin IP (Figure 2B) is not convincing given the prominent band in the Ctr IP; the control IP should be performed with IgG mouse or rabbit instead of blank beads; and what happens to the mobility of Rad51 foci following gamma-tubulin and/or KIF2C disruption?

*Regarding the DSB-like dsDNA in vitro experiments, there are a number of weaknesses, and additional controls are necessary, especially since gamma-tubulin is highly sticky (non-specifically),

© 2025. Published by The Company of Biologists under the terms of the Creative Commons Attribution License (https://creativecommons.org/licenses/by/4.0/).

and so there is no evidence that it is not an artifact. First, can different dsDNA sequences with free ends pull down the gamma-Tub and repair proteins? Is this ability lost if the ends of the dsDNA molecule are not free? Is gamma-Tub, but not alpha-Tub or beta-Tub, pulled down with the free-ended dsDNA?

*Regarding the immunoprecipitation and proteomic analysis, the description of the approaches and datasets are missing from the materials and methods section. In addition, the proteomic data related to Figure 2A should be shown in a more complete and informative manner, illustrating the total peptide counts, percent coverage of the amino acid sequence, replicate information, and statistical confidence (FDR or similar). The authors should also depict the data in a more visually informative way. One additional possibility is to also show the enriched proteins grouped by cellular function/pathway and visually depict their relative enrichment.

*The quality of the co-immunoprecipitation blots (Figures 2B and C) could be improved. Firstly, the input for Ku80 is not even between the control and Bleomycin lanes in Figure 2B and this blot has a higher background compared to the other blots. The gamma-tubulin blot has very messy input lanes. The authors don't specify their % input, but they may want to consider using a lower % input to help visualize the bands in the IP lanes (especially in Figure 2C).

*The knockdown of gamma-tubulin is inconsistent and, in certain cases, not ideal. In Figure 3C, it's unclear why the 6-hour time point has almost 100% knockdown whereas the other lanes do not have nearly as good a knockdown.

*The authors only use western blot of gamma-H2Ax to quantify DNA damage, which does not provide information at a single cell level. Adding single-cell imaging data (e.g., foci number vs. size) for a sufficiently high number of cells across the cell population would provide a higher level of resolution for the data measuring DNA damage, and would better assess claims related to DSB clustering and colocalization with gamma-tubulin and KIF2C. Similarly, using immunofluorescence would provide a more sensitive single-cell method of measuring DSB kinetics. Such single-cell analyses would especially better reveal whether the herein-reported processes operate broadly across the cell population.

*The authors should include live-cell imaging experiments to better understand the kinetics of gamma-tubulin recruitment to the sites of damage. Such experiments can also help visualize DSB mobility and how it is impaired upon tubulin knockdown.

*It's unclear why the authors treated cells with 20 Gy of IR, which is a remarkably high level of damage. 53BP1 foci can be visualized with lower IR amounts, such as 5 Gy. Also, the 53BP1 foci do not look well-defined in Figure 5C, which may be due to the extremely high amount of damage induced by 20 Gy IR.

*The study does not have any cell cycle controls. The authors should show how gamma-tubulin knockdown affects the cell cycle, which is an important consideration for DSB repair, especially by HR. The authors should also use of cell cycle markers such as EdU to assess how gamma-tubulin impacts DSB repair in the different cell cycle phases.

Other comments:

*There is a typo in Figure 2C; should be KU80, not KU86.

*In Fig. 5A, how was the distance traveled calculated exactly? Is the movement calculated via MSD or is it the distance separating the start and end position of the DSB? This should be better described in the results section or within the figure panel/legend.

*Overall, the methods section was often incomplete and will require careful revision.

*In the growth assay (Figure 6), the y-axis label should reflect the normalization used. Also, it's unclear why the control cell growth in B is different than A, C and D when all the experiments were done in HeLa cells.

© 2025. Published by The Company of Biologists under the terms of the Creative Commons Attribution License (https://creativecommons.org/licenses/by/4.0/).

*The study has some promising growth data in cell lines and organoids. In addition to the in vitro work, the authors should consider adding in vivo experiments using gamma-tubulin inhibitors in conjunction with chemotherapeutics to assess whether the in vitro results can be recapitulated in vivo. This would strengthen the therapeutic relevance of this study. Alternatively, the authors could at least acknowledge that in vitro and in vivo data may be quite different and would require future study. Similarly, given that gamma-tubulin and PARP1 interact, it would be interesting to see the effect of combining gamma-tubulin inhibition with PARP inhibition in HR-deficient cells.

First revision

Author response to reviewers' comments

Reviewer 1 Advance Summary and Potential Significance to Field:
The manuscript demonstrates the role of g-tubulin during the repair of double strand breaks (DSBs) in human cells. The topic is very timely and extremely interesting. The authors clearly show that a fraction of g-tubulin is recruited to DNA damage in the nucleus and colocalize with several repair factors such as PAR, ATM, 53BP1 and Polymerase l. Although the precise function of g-tubulin during DNA repair is not totally elucidated, they show that depletion of g -tubulin impedes DNA repair both by homologous recombination and non-homologous recombination. In addition, g-tubulin is implicated in the higher mobility of 53BP1 repair foci, which provokes the clustering of 53BP1 focus. Considering the important role of g-tubulin, they finally show that inhibitors of g-tubulin enhance the cytotoxic effect of DNA damaging agents.

The manuscript is very well written, clear and concise. The results clearly indicate the recruitment of g-tubulin at DNA lesions and its importance for DNA repair. Some results need to be clarified. For example, it would important to show the kinetics of g-tubulin recruitment at DNA lesions, the liquid properties of 53BP1 foci and the way they interact with g-tubulin could be discussed, the results on Rad51 are not very convincing, replicates of IP experiments should be shown, mean square displacements curves of 53BP1 should be shown and fitted.

In short, the manuscript is very interesting, but certain results need some clarifications.
Response: We thank the reviewer for the positive remarks, and have addressed the concerns, as explained below.

Reviewer 1 Comments for the Author:
The manuscript is very interesting but several points should be clarified and discussed.

To induce DNA damage on cells, the authors used 405 nm laser micro-irradiation under a Zeiss Axiovert 200M Microscope. Do they pre-sensitize the cells before irradiation? If they don't pre sensitize the cells, what kind of DNA damages are induced with the 405 nm laser? What is the laser power at the sample in W per cm2?
After micro-irradiation, they observe a colocalization of g-tubulin with PAR and other repair factors. The time after micro-irradiation at which the IF was performed needs to be indicated. It is known that PAR is recruited just a few seconds after damage, and is disassembled 10 minutes after damage. The data in Figure 2C suggests that g-tubulin is recruited very quickly after irradiation.
It would very informative to perform a timelapse of the g-tubulin recruitment after micro-irradiation in live cells.
Response: As the reviewer requested, we have provided more details to clarify the laser microirradiation experiment in Figure Legends and Materials and Methods, and included new data to strength the conclusion.
Our initial laser microirradiation did not use pre-sensitization. This laser system delivered relatively high energy density (10^6 W/cm2 per our calculation) to induce DNA damage. Laser microirrdiation typically induce a mixture of various DNA lesions. However, we confirmed the

© 2025. Published by The Company of Biologists under the terms of the Creative Commons Attribution License (https://creativecommons.org/licenses/by/4.0/).

efficient induction of DSBs using this system, e.g., using GFP-Ku70, GFP-polymerase lambda, and other DSB repair markers (review figure shown here).

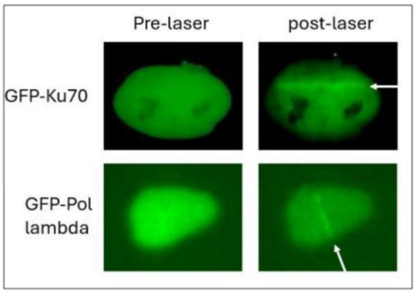

Inspired by the reviewer's comment, we decided to confirm this finding, using a different imaging and laser system. This system uses Hoechst 3342 for pre-sensitization. We were able to confirm that gamma-tubulin was recruited to damage site, similar to GFP-polymerase lambda, a known DSB responder (Fig. 1D & supplemental Fig. S1D). We have included this new result in the revised manuscript. We have also included a timelapse for the gamma-tubulin recruitment (supplemental Fig. S1E). The new laser micro-irradiation was imaged under Zeiss 880 confocal laser scanning microscope.

Figure 2, is it possible to quantify the immune-precipitation bands ?
Figure 2B, for PARP1 and especially for Rad51, there is a clear band in the absence of bleomycin. The authors mention that there is "a notable level of g- tubulin-Rad51" association, albeit accompanied by a lower non specific signal". Is it simply an artefact or is there non-specific interaction of Rad51 and PARP1 with g-tubulin in the absence of damage?
Response: As the reviewer requested, we have performed additional IP experiments, replaced the representative data (Fig. 2B), and provided quantification (Fig. 2C-2E). Results showed consistent associations between gamma-tubulin and DNA repair proteins in response to DNA damage.

In figure 4, the authors use I-SceI-induced HR or NHEJ reporter cells. Can they precise the timing and the efficiency of the I-SceI cutting in these cell lines?
Response: As the reviewer requested, we provided more information about the I-SceI-induced NHEJ assay, in the new "DNA repair assays" section in Materials and Methods. The efficiency of I- SceI expression was confirmed by both the NHEJ/HR induction and immunoblotting of HA-I-SceI, as in our previous study (Elife . 2020 Jan 17:9:e53402. doi: 10.7554/eLife.53402.). This HA-I-SceI vector was well characterized in previous studies (Nat Methods. 2011 Jul 10;8(8):671-6. doi: 10.1038/nmeth.1648). Additional experiment information is provided in the Materials and Methods and Figure Legends.

Figure 5, the authors measure the mobility of 53BP1 foci in living cells. For that, they use a U2OS cell line expressing EGFP-53BP. However, there is no mention of this cell line in the material and method. Is it an endogenous fusion of EGFP to 53BP1? is it a stable cell line expressing EGFP-53BP1, or is it a transitory transfection of a plasmid? Several studies showed that 53BP1 proteins form membrane less condensates via liquid liquid phase separation (doi: 10.15252/embj.2018101379; https://doi.org/10.1038/s41586-023-06635-y).
Changing the physiological level of proteins expression might change the condensate properties. It is thus, essential to favor experiments using endogenous tagged proteins or to carefully check the level of expression by Western Blot.
In figure 5A, the authors analyze the mobility of 53BP1 foci: can they show a typical trajectory in WT versus siRNA treated cell ? Can they show the mean square displacement and fit the curve to extract diffusive parameters ?
When the author use an Apple-53BP1trunc plasmid, can they explain what it is 53BP1trunc and is it overexpressed ?
Response: As the reviewer requested, we have provided additional information and new data to clarify these questions.

© 2025. Published by The Company of Biologists under the terms of the Creative Commons Attribution License (https://creativecommons.org/licenses/by/4.0/).

We used transient expression of EGFP-53BP1 for the foci mobility study. In the revised manuscript, we included new data to monitor the level of 53BP1 expression. We believe that the expression is at a physiological level, only moderately upregulated from the endogenous level (supplementary Figure S8).
As the reviewer requested, we have provided additional measurement of the mean squire displacement and foci trajectory (Figure 5A & 5B). These new panels are consistent with, and support the previous measurement and quantification of distance traveled by individual 53BP1 foci (Figure 5C).
More information about the 53BP1-trunc vector is provided (amino acids 1220-1711, and corresponding reference) in Materials and Methods.

Discussion:
In the discussion, the physical nature of 53BP1 should be discussed. Do foci look spherical or not? in Oshidari 2020, Rad52 droplets exhibit wetting behavior against MT. Do they see this phenomenon here for 53BP1 against tubulin? The role of AR2/3 should be mentioned as well.
Response: We included these studies and references in the discussion in the revised manuscript. While our study did not address the potential wetting behavior of 53BP1 and other DSB proteins, their physical nature, in relationship with tubulins, is interesting. Future studies, building on findings of ours and others, may reveal more insights.

Minor comments:
Replicates of IP and IB should be shown in supplementary data.
In the material and methods, they authors need to provide de references of all their anti-bodies against g-tubulin, ATP, PAR, 53BP1 polymerase l.
Have they tried anti bodies against Rad51 and if yes, what do they see?

Response: As requested by the reviewer, additional information is provided for the antibodies used in the new Immunoprecipitation section in Materials and Methods. The IP experiments were re- performed, with new results added in the revised manuscript (Fig. 2B), and quantifications from multiple independent experiments provided (Fig. 2C-2E).

When they use bleomycin of X rays, can they give an idea of the type of damage and the number of breaks induced? For example, 20 Gy of X rays seems very high for human cells.
Response: Bloemycin and X rays are known to induce DSBs, along with other types of DNA damage. We show in this study that gamma-tubulin associates with DSB-mimicking, binds to DSB repair proteins, and mediates DSB repair. 20 Gy X ray does seem high, but the dose was chosen because it induced DSBs (gamma-H2AX), and the induced DSBs were largely repaired within hours. Thus, this dose is appropriate based on the biological effect. It is possible that the small benchtop X-ray irradiator administered lower energy density. We have included this clarification about dose consideration in the revised manuscript.

Reviewer 2 Advance Summary and Potential Significance to Field:
The authors present data suggesting a nuclear role for gamma-tubulin in DNA double-strand break (DSB) repair in human cells. The data potentially suggest that gamma-tubulin is recruited to DNA damage sites, promotes the targeting of repair proteins (NHEJ and HR factors) to the chromatin fraction of cells exposed to DNA damage, and mediates repair by NHEJ and HR. The authors observed that gamma-tubulin is required for DSB mobilization and potentially works in the same pathway as KIFC2, a microtubule-depolymerizing kinesin. A tentative mechanism is proposed where somehow nuclear gamma-tubulin works in concert with KIFC2 to mediate DSB mobilization and clustering, which would, in turn, somehow increase the local concentration of repair factors, thereby increasing accurate repair and cell survival. The authors also suggested a potential therapeutic avenue of further sensitizing cancer cells to irradiation or chemotherapeutics by pharmacologically inhibiting gamma-tubulin. The authors do acknowledge late in the discussion that various tubulin isoforms and microtubules could promote repair by operating via different mechanisms, including through the cytoskeleton exerting forces onto chromatin by pushing onto the nuclear envelope.

Overall, investigating the role of tubulin in DNA double-strand break repair and mobility is a

© 2025. Published by The Company of Biologists under the terms of the Creative Commons Attribution License (https://creativecommons.org/licenses/by/4.0/).

topical area in the DNA repair field and this study may demonstrate that nuclear gamma-tubulin is involved in human DNA repair. Although the study is of potential interest, major revisions are necessary.
Response: We thank the reviewer for the positive remarks. We have addressed the concerns, as explained below.

Reviewer 2 Comments for the Author:
Major comments:

**Mechanistically, the study does not provide a clear and well-supported mechanism through which gamma-tubulin promotes DSB repair. Although gamma-tubulin appears to potentially impact DSB mobility and the recruitment of NHEJ and HR factors to chromatin, it is unclear how these processes are occurring. The authors should consider the following questions:*
-How is the entire process triggered upon DSB induction? For example, do the DNA Damage Response kinases ATM, ATR, and/or DNAPK increase the nuclear localization and/or chromatin association of gamma-Tub?
-How might gamma-tubulin promote the recruitment of both NHEJ and HR factors?
-Does gamma-tubulin interact with KIF2C? Is gamma-tubulin required for KIF2C-mediated DSB repair?
-Is the role of gamma-tubulin related to or distinct from the role of alpha/beta-Tubulin? considering the better-documented roles of alpha/beta-tubulin in DNA repair, it is also important to assess whether the disruption of gamma-tubulin disrupts alpha/beta-tubulin-dependent DSB repair as well.
-Clustering is not generally viewed as a repair-promoting mechanism. Rather, it is a process primarily affecting hard-to-repair or persistent breaks. So, it is very unlikely that a potential increase in DSB clustering would be a primary mechanism to drive physiologically relevant DNA repair events.
-Previous studies have provided evidence that tubulin/microtubules mediate DNA repair in different organisms via various nuclear and cytoplasmic mechanisms, which are generally mentioned although not accurately discussed or cited in the introduction and discussion sections. The authors should better explain how their proposed mechanism intersects with these and other studies in the literature. Especially, the mechanism proposed here, although still not supported by the data, seems illogical. How can a mechanism promoting DSB clustering as a primary function serve to maintain accurate DSB repair and preserve genome stability and viability in the long term?

Response: We appreciate the reviewer's insights about the underlying mechanisms. Our study reported a new role of gamma-tubulin in mediating DSB repair, and revealed a mechanism via which gamma-tubulin mediates DSB mobility and foci formation, and facilitates the recruitment of NHEJ and HR factors to chromatin.

The points raised by the reviewer are now carefully discussed in the revision, with the acknowledgement that future work is needed to better understand the process, as well as to discover additional mechanistic insights.

Inhibition of ATM, ATR, or DNA-PK did not prevent gamma-tubulin recruitment to laser-induced DNA damage sites. We have included this new data in the revised manuscript (Supplementary Fig. S2).
This does suggest independent mechanisms of gamma-tubulin recruitment that remain to be investigated in future studies.
The fact that gamma-tubulin facilitates the chromatin recruitment of both NHEJ and HR factors after DNA damage at least partially explains why it promotes both HR and NHEJ. As in our model (Fig. 7), the cluster of DSB sites into micronuclear domains can facilitate the local recruitment of these repair factors.
Our data suggest that gamma-tubulin and Kif2C function coordinately in DNA repair. More detailed investigation in the functional interplay between these factors, and other MT components is interesting, but shall be pursued in future studies.
We did not observe convincing recruitment of alpha/beta-tubulin to laser-induced DNA damage sites (data not shown), even though these tubulins were associated with DSB mimicking DNA template in vitro (now shown in supplementary Fig. S1A). MT poisons which

© 2025. Published by The Company of Biologists under the terms of the Creative Commons Attribution License (https://creativecommons.org/licenses/by/4.0/).

target alpha/beta- tubulin suppressed DSB mobility and repair, suggesting a direct or indirect role of these tubulins (Cell . 2015 Nov 5;163(4):880-93. doi: 10.1016/j.cell.2015.09.057.). The precise role of alpha/beta- tubulin in DNA repair is interesting, but beyond the scope of this study. Here, our finding revealed a new role of gamma-tubulin in DSB repair, and provided proof-of-principle for therapeutic targeting of gamma-tubulin in cancer. Compared to alpha/beta tubulin, gamma-tubulin is not the building element of MT, and its targeting in cancer is not adequately explored.
Finally, data from us and others suggest that clustering of DNA damage and the formation of repair centers (nuclear foci) occur quickly after DNA damage, and is responsive to low dose, repairable level, of DNA damage (Proc Natl Acad Sci U S A . 2012 Jan 10;109(2):443-8; Curr Opin Cell Biol . 2004 Jun;16(3):328-34). Even though persistent DNA damage foci may be associated with sustained unrepaired DNA damage, as the reviewer pointed out, the general mechanism of DNA damage mobility and clustering into repair center are likely also important for the initial repair.
**Is the herein-studied role of nuclear gamma-tubulin distinct from its role in nucleating microtubules at the centrosome and other MTOCs? Does nuclear gamma-tubulin nucleate or prevent the nucleation of short microtubules in the nucleus upon DNA damage induction? How is this intersecting with the role of KIF2C as a microtubule-depolymerizing kinesin? Moreover, the centrosome can harbor various DNA repair proteins (see review Mullee and Morrison, 2015). Is it possible that the mechanism suggested in this manuscript relates to changes in the centrosome upon DNA damage?*
Response: We appreciate the point raised by the reviewer here. Gamma-tubulin can influence DNA repair indirectly via centrosome regulation, as the reviewer indicated. Our study does not rule out this possibility. We included this possibility in the discussion section of the revised manuscript.
However, in this study, we showed direct recruitment of gamma-tubulin to the sites of nuclear DNA damage, association of gamma-tubulin with DSB repair proteins, and its role in promoting DSB repair. Together, we reveal a new role of gamma-tubulin beyond its conventional cytoplasmic and MTOC functions

**A substantial proportion of the data provided in this study is not of a high-quality, leaving this reviewer with the impression that many claims are not supported by the data presented. There are a number of issues regarding the imaging data provided:*
-In Figure 1, there is a lot of background in the images, especially in the gamma-tubulin red channel. Gamma-tubulin is typically enriched in the centrosome with minimal cytoplasmic staining. The images provided raise questions about the quality and specificity of the antibodies/imaging. An inverted fluorescence microscope was used to capture the images used in this study. A confocal microscope and 3D images might provide higher-resolution 3D. Additionally, knockdown controls assessing whether the weak nuclear gamma-Tubulin signals reported are indeed abolished upon gamma-Tubulin knockdown are missing and sorely needed.
Response: We improved data quality in the revised manuscript. In Figure 1, additional imaging data are provided, using a confocal microscope (Fig. 1C, 1D, supplementary Fig. S1D, S1E). A different laser micro-irradiation system with pre-sensitization was used to confirm the DNA damage recruitment of gamma-tubulin (Fig. 1). The specificity of the antibody for gamma-tubulin IF is confirmed by siRNA (supplementary Fig. S1C). The localization of gamma-tubulin is consistently seen also with direct fluorescence of recombinant gamma-tubulin.

-In Figure 1C, it appears that the left cell was irradiated along the diameter of the nucleolus, resulting in a strange pattern in PARP recruitment (i.e., at the periphery of the nucleolus). Is that the case? Please clarify either way.
-None of the imaging figures are accompanied by quantitative data. For example, colocalization can be accompanied by intensity profile graphs. Moreover, the gamma-tubulin ‘foci’ depicted in Figure 1D should be accompanied by quantifications that compare control and DNA damage conditions on a single-cell and cell population level.
-In Figure 1E, it is difficult to see the recruitment of mCherry gamma-tubulin to the sites of damage. In fact, these data suggest that the protein is not recruited to the site of damage, raising doubt about the overall model presented.
-There are no scale bars on any of the images provided.

© 2025. Published by The Company of Biologists under the terms of the Creative Commons Attribution License (https://creativecommons.org/licenses/by/4.0/).

Response: The quality of these imaging data is now improved.
Additional imaging data are provided, using a confocal microscope and a distinct laser system (Fig. 1C, 1D, supplementary Fig. S1D, S1E). The specificity of the antibody for gamma-tubulin IF is confirmed by siRNA (supplementary Fig. S1C). The localization of gamma-tubulin is consistently seen also with direct fluorescence of recombinant gamma-tubulin.
Area of laser microirradiation is denoted by a white arrow (Fig. 1B, 1D, supplemental S1D) or white line (supplemental Fig. S1B). Quantitative graph is provided to confirm the colocalization (Fig. 1C, supplemental Fig. S1E). Foci colocalization of gamma-tubulin and 53BP1 is shown in Fig. 1E and supplementary movies 1-4. Notably, 53BP1 foci are induced by DNA damage, and do not present under normal conditions (J Cell Biol. 2000 Dec 25;151(7):1381–1390). Scale bars are included in Fig. 1D, 1E, 5B, 5F, and supplementary Fig. S1B, S1D, S2, S5A, S8.

**In Figure 1, what is occurring to gamma-tubulin upon DSB induction? Specifically, the authors should show whether the levels of gamma-tubulin change overall within the same experimental conditions (e.g., with a western blot) and whether the nuclear vs. cytoplasmic localization of gamma-tubulin changes via a nuclear fractionation. Additionally, it appears that the levels of gamma-tubulin do change upon DNA damage in certain figures (e.g., Figure 4C and first two lanes) but do not change in other figures (e.g., Figure 4D). How might the authors explain this discrepancy?*

Response: While there were some experimental variations, we concluded from multiple independent experiments that gamma-tubulin expression is not significantly induced after DNA damage. However, its nuclear localization is elevated following DNA damage. Representative data and quantification are shown in the revised manuscript (Fig. 1F & 1G).

**The authors claim a substantial role for gamma-tubulin in promoting the recruitment of key DSB repair proteins (NHEJ and HR) to chromatin. To this end, a series of chromatin fractionations are shown (Figure 4), but the data are not very convincing. For example, in Figure 4F, there is only a very slight reduction in Rad51's recruitment to chromatin following gamma-tubulin knockdown, despite gamma-tubulin disruption strongly decreasing the repair of HR and NHEJ reporters. In fact, the overall data assessing links between gamma-tubulin and HR repair is weak. Specifically, no HR- related protein was shown in the dsDNA IP (Figure 1A) as a positive control and no HR protein appeared in the proteomic analysis (Figure 2A); the blot for Rad51 in the gamma-tubulin IP (Figure 2B) is not convincing given the prominent band in the Ctr IP; the control IP should be performed with IgG mouse or rabbit instead of blank beads; and what happens to the mobility of Rad51 foci following gamma-tubulin and/or KIF2C disruption?*
Response: We showed that gamma-tubulin promoted the chromatin recruitment of NHEJ and HR proteins after DNA damage. This was seen consistently in independent experiments (as quantified in Fig. 4G).
As the reviewer pointed out, our mass spec experiment did not identify HR factors. However, we performed additional IP experiments to investigate the association between gamma-tubulin and DSB repair factors. As shown in the revised Fig. 2B-2F, we were able to confirm associations between gamma-tubulin and Ku80, PARP1, Rad51, RPA70, RPA32, and Artemis. This new result strengthened the connection between gamma-tubulin and core NHEJ/HR factors. The associations are specific, not evident in the blank control (as in Fig. 2) or using other gamma-tubulin antibodies which did not IP gamma-tubulin (data not shown). As discussed in the revised manuscript, future studies are needed to further investigate the molecular and functional details of these protein associations, including Rad51 foci mobility.

**Regarding the DSB-like dsDNA in vitro experiments, there are a number of weaknesses, and additional controls are necessary, especially since gamma-tubulin is highly sticky (non- specifically), and so there is no evidence that it is not an artifact. First, can different dsDNA sequences with free ends pull down the gamma-Tub and repair proteins? Is this ability lost if the ends of the dsDNA molecule are not free? Is gamma-Tub, but not alpha-Tub or beta-Tub, pulled down with the free-ended dsDNA?*

© 2025. Published by The Company of Biologists under the terms of the Creative Commons Attribution License (https://creativecommons.org/licenses/by/4.0/).

Response: The in vitro pulldown using DSB-like dsDNA did have its limitation, as the reviewer pointed out. Thus, it is important that we were able to confirm the DNA damage recruitment of gamma-tubulin in cells using multiple imaging assays.
The control pull-down using blank beads did not recover gamma-tubulin. On that note, alpha/beta tubulins are stickier, and seen in the control pulldown too, albeit clear increase in the DSB-like groups (supplemental Fig. S1A). We were unable to use a circular DNA (without DSB ends) because we needed to conjugate biotin to one end of the DNA.
We were able to confirm the in vitro DSB-binding of gamma-tubulin using two independent substrates. The initial mass spec identification was performed using a poly A-T 70 mer double strand oligonucleotides (supplementary Fig. S1A). The confirmatory pulldown was performed using a 500 bp double stranded DNA fragments with a different sequence (Fig. 1A). In both cases, gamma-tubulin was associated with DSB-mimicking templates but not control beads.

**Regarding the immunoprecipitation and proteomic analysis, the description of the approaches and datasets are missing from the materials and methods section. In addition, the proteomic data related to Figure 2A should be shown in a more complete and informative manner, illustrating the total peptide counts, percent coverage of the amino acid sequence, replicate information, and statistical confidence (FDR or similar). The authors should also depict the data in a more visually informative way. One additional possibility is to also show the enriched proteins grouped by cellular function/pathway and visually depict their relative enrichment.*
Response: As suggested by the reviewer, additional experimental details are provided in the revised manuscript, in the immunoprecipitation section in Materials and Methods. Furthermore, more information about the proteomic identification of PARP1 and DNA-PKcs in gamma-tubulin IP are included in the revised manuscript (Fig. 2A).

**The quality of the co-immunoprecipitation blots (Figures 2B and C) could be improved. Firstly, the input for Ku80 is not even between the control and Bleomycin lanes in Figure 2B and this blot has a higher background compared to the other blots. The gamma-tubulin blot has very messy input lanes. The authors don't specify their % input, but they may want to consider using a lower % input to help visualize the bands in the IP lanes (especially in Figure 2C).*
Response: As suggested by the reviewer, new and improved IP data are included in the revised manuscript (Fig. 2B), with more experimental details provided in Materials and Methods and Figure Legends. Additional quantifications from multiple independent experiments are also shown (Fig. 2C-2E).

**The knockdown of gamma-tubulin is inconsistent and, in certain cases, not ideal. In Figure 3C, it's unclear why the 6-hour time point has almost 100% knockdown whereas the other lanes do not have nearly as good a knockdown.*
Response: As suggested by the reviewer, better quality data with more consistent knockdown efficiency are provided in the revised Fig. 3C. Additional supplementary data are included to confirm the same conclusion that gamma-tubulin suppression impaired DNA repair (supplementary Fig. S4, S5, & S6).

**The authors only use western blot of gamma-H2Ax to quantify DNA damage, which does not provide information at a single cell level. Adding single-cell imaging data (e.g., foci number vs. size) for a sufficiently high number of cells across the cell population would provide a higher level of resolution for the data measuring DNA damage, and would better assess claims related to DSB clustering and colocalization with gamma-tubulin and KIF2C. Similarly, using immunofluorescence would provide a more sensitive single-cell method of measuring DSB kinetics. Such single-cell analyses would especially better reveal whether the herein-reported processes operate broadly across the cell population.*
Response: As suggested by the reviewer, single cell measures of DSB, using 53BP1 foci formation, are included in the revised manuscript (supplementary Fig. S5). Consistent data are shown for both the induction of endogenous DNA damage and the effect on DNA repair.

**The authors should include live-cell imaging experiments to better understand the kinetics of gamma-tubulin recruitment to the sites of damage. Such experiments can also*

© 2025. Published by The Company of Biologists under the terms of the Creative Commons Attribution License (https://creativecommons.org/licenses/by/4.0/).

help visualize DSB mobility and how it is impaired upon tubulin knockdown.
Response: As suggested by the reviewer, improved imaging data are shown for gamma-tubulin recruitment to DNA damage sites (Fig. 1D and supplemental Fig. S1D), with quantification and kinetics (Fig. 1C and supplemental Fig. S1E). The conclusion was also confirmed using a different laser-system and confocal microscopy.

**It's unclear why the authors treated cells with 20 Gy of IR, which is a remarkably high level of damage. 53BP1 foci can be visualized with lower IR amounts, such as 5 Gy. Also, the 53BP1 foci do not look well-defined in Figure 5C, which may be due to the extremely high amount of damage induced by 20 Gy IR.*
Response: 20 Gy X ray does seem high, but the dose was chosen because it induced DSBs (gamma-H2AX), and the induced DSBs was largely repaired within hours. Thus, this dose is appropriate based on the biological effect. It is possible that the small benchtop X-ray irradiator administered lower energy density. We have included this clarification in the revised manuscript. 53BP1 foci presentation was also improved in the revised manuscript.

**The study does not have any cell cycle controls. The authors should show how gamma-tubulin knockdown affects the cell cycle, which is an important consideration for DSB repair, especially by HR. The authors should also use of cell cycle markers such as EdU to assess how gamma-tubulin impacts DSB repair in the different cell cycle phases.*
Response: As suggested by the reviewer, we examined the impact of gamma-tubulin in cells synchronized in G1 or S phase. In both phases, gamma-tubulin knockdown induced gamma-H2AX accumulation, suggesting its role in DNA repair in both phases (supplementary Fig. S3). This is probably expected as we showed gamma-tubulin mediated both NHEJ and HR.

Other comments:

**There is a typo in Figure 2C; should be KU80, not KU86.*
Response: This is corrected.

**In Fig. 5A, how was the distance traveled calculated exactly? Is the movement calculated via MSD or is it the distance separating the start and end position of the DSB? This should be better described in the results section or within the figure panel/legend.*
Response: The distance travelled was calculated from the total cumulated distance travelled by foci at each time lapse recording within the 10-min time period. In the revised manuscript, we have provided additional measurement of the mean squire displacement (Figure 5A & 5B). These new panels are consistent with, and support the previous measurement and quantification of distance traveled by individual 53BP1 foci (Figure 5C).
More detailed information about these measurements has been added to the manuscript, within the Materials and Methods (Microscopic analysis of DNA damage foci formation and mobility) and figure legends.

**Overall, the methods section was often incomplete and will require careful revision.*
Response: We have updated the Materials and Methods section.

**In the growth assay (Figure 6), the y-axis label should reflect the normalization used. Also, it's unclear why the control cell growth in B is different than A, C and D when all the experiments were done in HeLa cells.*
Response: We have changed the Y-axis label as requested. The growth rate may appear different in different experiments due to the level of cell confluency, passages of cells, and types of cell line, etc.

**The study has some promising growth data in cell lines and organoids. In addition to the in vitro work, the authors should consider adding in vivo experiments using gamma-tubulin inhibitors in conjunction with chemotherapeutics to assess whether the in vitro results can be recapitulated in vivo. This would strengthen the therapeutic relevance of this study. Alternatively, the authors could at least acknowledge that in vitro and in vivo data may be quite different and would require future study. Similarly, given that gamma-tubulin and PARP1 interact, it would be interesting to see the effect of combining gamma-tubulin inhibition with PARP inhibition in HR-deficient cells.*

© 2025. Published by The Company of Biologists under the terms of the Creative Commons Attribution License (https://creativecommons.org/licenses/by/4.0/).

Response: We agree with the reviewer that further investigations of gamma-tubulin targeting in cancer therapy, using in vivo models, and in combination with PARPi, are interesting. We believe that they shall be pursued in future studies, with our current study revealed the new role of gamma- tubulin in DNA repair and provided the proof-of-principle of its therapeutic targeting. We have included these discussions in the revised manuscript.

Second decision letter

MS ID#: JOCES/2024/262255

MS TITLE: Gamma-tubulin Mediates DNA Double Strand Break Repair

AUTHORS: Abhishikt David Solomon, Odjo G Gouttia, Ling Wang, Songli L Zhu, Feifei Wang, Yanqiu Li, Mohammadjavad Paydar, Tadayoshi L Bessho, Benjamin Kwok, and AIMIN PENG

I am happy to tell you that your manuscript has been accepted for publication in Journal of Cell Science, pending standard publication integrity checks. Please do take note of the few small comments from the referee, which we would encourage you to address in your final manuscript.

Reviewer 1

Advance summary and potential significance to field

The authors have addressed most of the points address in the 2 reviewers. Several new experiments have been performed. As suggested by the reviewers, some data have been better quantified. The text is also more precise and several references have been added.

Comments for the author

Figure 1E: the authors should be carrefull about imaging 53BP1 by IF, as fixation can strongly alter liquid liquid phase separation.
https://elifesciences.org/articles/85671 The authors need to include a note about this and always perform an experiment in living cells in parallel. 53BP1 have been imaged already by other teams in living cells (https://pubmed.ncbi.nlm.nih.gov/29419809/
https://www.embopress.org/doi/full/10.15252/embj.2018101379 ....), so there is specific need for this manuscript to show it again. However, it is important to refer to these papers and to mention that IF and imaging in live cells are similar in the case of 53BP1, which is not the case for all types of LLPS.

One comment is not addressed: “Have you tried anti-bodies against Rad51 and if yes, what do they see” ?
Maybe the question was not clear: have you tried anti-bodies against Rad51 in cells to performed IF and looked by microscopy ? If yes, do you observe foci filaments, both ?

In the sentence "An interesting study in yeast showed that the lipid droplets formed ...", do you mean "liquid droplets" adn not "lipid droplets" ?

© 2025. Published by The Company of Biologists under the terms of the Creative Commons Attribution License (https://creativecommons.org/licenses/by/4.0/).
